# Standardized Microplate‐Based Assays for Quantifying Antioxidant Activity in Extracellular Vesicles and Nanovesicles Across Biological Kingdoms

**DOI:** 10.1002/jev2.70392

**Published:** 2026-11-03

**Authors:** Juan Antonio Nieto, María‐Carmen López de las Hazas, Victoria Martín‐Santiago, Laura Rubio‐Silvan, Carmen Mazarío‐Gárgoles, Ludovica Attanasio, Benedetta Mercuriali, João Tomé‐Carneiro, Giulia Bernabè, Andrea del Saz‐Lara, Carmen Crespo, María Ángeles Ávila‐Gálvez, Emma Burgos‐Ramos, Juan Carlos Espín, Andrea Chini, Alberto Dávalos

**Affiliations:** ^1^ Laboratory of Epigenetics of Lipid Metabolism Instituto Madrileño de Estudios Avanzados (IMDEA)‐Nutrición, CEI UAM+CSIC Madrid Spain; ^2^ Bioactivity and Nutritional Immunology Group (BIOINUT), Faculty of Health Science Universidad Internacional de Valencia (VIU) Valencia Spain; ^3^ Department of Nutrition and Metabolism, Institute of Food Science, Technology and Nutrition Spanish National Research Council, CSIC‐ICTAN Madrid Spain; ^4^ Department of Pharmaceutical Sciences Università Degli Studi di Milano Milan Italy; ^5^ Laboratory of Functional Foods Instituto Madrileño de Estudios Avanzados (IMDEA)‐Nutrición, CEI UAM+CSIC Madrid Spain; ^6^ Department of Molecular Medicine University of Padova Padova Italy; ^7^ Facultad de Ciencias de la Salud Universidad Tecnológica Atlántico Mediterráneo ‐ UTAMED Málaga Spain; ^8^ Laboratory of Food & Health, Research Group on Quality, Safety, and Bioactivity of Plant Foods, CEBAS‐CSIC Campus Universitario de Espinardo Murcia Spain; ^9^ Nutraceuticals and Neuroendocrine and Neurodegenerative Diseases Group (NUENE), Institute of Biomedicine. Faculty of Environmental Sciences and Biochemistry University of Castilla‐La Mancha Toledo Spain; ^10^ Plant Molecular Genetics Department, National Centre for Biotechnology (CNB) Consejo Superior de Investigaciones Cientificas (CSIC) Madrid Spain; ^11^ Consorcio CIBER de la Fisiopatología de la Obesidad y Nutrición (CIBERObn) Instituto de Salud Carlos III (ISCIII) Madrid Spain

**Keywords:** ABTS, antioxidant, bacterial EVs, extracellular vesicles, ORAC, phenolic compounds, plant‐derived nanovesicles

## Abstract

The antioxidant activity of extracellular vesicles (EVs) and nanovesicles are increasingly linked to their physiological and therapeutic functions, yet its accurate quantification remains limited by low EV yield, co‐isolated contaminants and the lack of standardized methodologies. Here, we establish optimized microplate‐based protocols for ABTS and ORAC assays specifically adapted to highly purified EVs and nanovesicles obtained by ultracentrifugation followed by size‐exclusion chromatography (UC + SEC). By minimizing sample input, implementing protein‐based normalization (TEAC/g EV protein), and introducing assay‐specific controls—including a self‐blank correction for ORAC—we achieve sensitive and reproducible measurements across plant‐, human plasma‐ and bacterial‐derived EVs and nanovesicles. Our results demonstrate that conventional isolation methods substantially overestimate antioxidant activity due to non‐vesicular contaminants, underscoring the need for rigorous purification. Using a detergent‐based disruption strategy compatible with ABTS, we further distinguish surface‐associated from cargo‐derived antioxidant activity, revealing a two‐tier functional organization of EVs. Notably, we report for the first time measurable antioxidant activity in bacterial extracellular vesicles (BEVs). Application of these methods to a human dietary intervention shows that EV antioxidant capacity is dynamically modulated in vivo. Collectively, this work provides a standardized and broadly applicable framework for the reliable assessment of EV and nanovesicles antioxidant properties across biological kingdoms.

## Graphical abstract

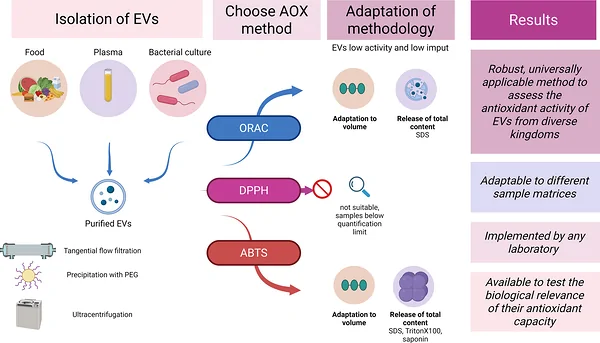

## 1 Introduction

Extracellular vesicles (EVs) are nanoparticles released by cells across all domains of life, acting as key mediators of intercellular communication across plants, animals and microorganisms. Increasing evidence indicates that EVs can modulate oxidative stress responses (Arslan et al. 2013; Liang et al. 2025; Xu et al. 2025; Zhang et al. 2024). Despite these observations, the intrinsic antioxidant activity of EVs themselves—independent of their ability to deliver antioxidant enzymes or regulatory RNAs—remains poorly defined and quantitatively uncharacterized (Luo et al. 2021).

A central barrier has been methodological: although ABTS, DPPH and ORAC assays are long established in microplate formats, they were originally developed for bulk extracts and have not been systematically adapted to the low yields, compositional complexity and stringent purity requirements characteristics of EV research. Previous studies reporting the antioxidant activity of plant‐derived EVs have employed single‐step isolation methods such as ultracentrifugation (UC) alone (Kilasoniya et al. 2023), sucrose density gradient (Gao et al. 2025) or polyethylene glycol (PEG) precipitation (Ma et al. 2024), usually alone, without subsequent purification steps that yield highly purified EVs, or expressed results using heterogeneous reporting units (Kilasoniya et al. 2023) and standards (Ma et al. 2024). Consequently, measured antioxidant activity in such studies is often dominated by contaminants, leading to substantial overestimation of true EV‐derived activity. Moreover, the reporting of results has been heterogeneous, with units ranging from percent inhibition to Trolox equivalents per millilitre or per gram of starting material, and with varying reference standards (Cusumano et al. 2025; Li et al. 2023).

Recent efforts have applied traditional radical scavenging assays to EVs, but without systematic optimization for vesicle‐specific constraints such as low abundance, protein normalization, or cargo encapsulation (Kilasoniya et al. 2023; Ma et al. 2024). Consequently, cross‐study comparisons are unreliable and mechanistic insights remain limited. No consensus framework currently exists for reproducible, biologically relevant quantification of EV antioxidant activity. This gap is particularly consequential as the field moves towards therapeutic applications of EVs, where functional characterization is essential for quality control, regulatory approval and clinical translation.

Here, we present an optimized microplate‐based ABTS and ORAC assays specifically adapted for EVs and plant‐derived nanovesicles (PDNVs). Our protocols minimize sample input, incorporate rigorous purification and normalization strategies based on protein (TEAC/g EV protein) to enable cross‐sample and cross‐study comparability. In addition, we have introduced a self‐blank correction for the ORAC assay to account for EV‐induced fluorescence quenching, an artifact previously overlooked in EV studies. We also develop and validate a membrane disruption approach based on detergent compatible with the ABTS assay that allows to differentiate surface‐associated from cargo‐derived antioxidant contributions.

We demonstrate broad applicability across biological kingdoms, including PDNVs and EVs, human plasma EVs and—for the first time—bacterial extracellular vesicles (BEVs). These methodological advances provide a standardized, reproducible and broadly applicable methodological framework for quantifying EV antioxidant properties, enabling reproducible measurements. These advances will enable meaningful cross‐study comparisons, facilitate mechanistic studies of EV‐mediated redox signalling and support the development of EV‐based therapeutics where antioxidant function is a key quality attribute.

## 2 Material and Methods

### 2.1 Chemicals and Reagents

Phosphate‐buffered saline (PBS, 1×), ABTS (2,2′‐azino‐bis(3‐ethylbenzothiazoline‐6‐sulfonic acid)), potassium persulfate, DPPH (2,2‐diphenyl‐1‐picrylhydrazyl), methanol, fluorescein disodium salt and AAPH (2,2′‐azobis(2‐amidinopropane) dihydrochloride) were purchased from Sigma‐Aldrich (Merck KGaA, Darmstadt, Germany) unless otherwise stated. Trolox (6‐hydroxy‐2,5,7,8‐tetramethylchroman‐2‐carboxylic acid) was used as an antioxidant standard. Ultrapure water was obtained from a Milli‐Q purification system (Millipore, Burlington, MA, USA).

### 2.2 Plant‐Derived Nanovesicle Isolation

Homogenized plant samples were sequentially centrifuged at 4°C to remove fibres, large particles and cellular debris: 5000 × g, 10,000 × g and 25,000 × g for 30 min each, followed by 35,000 × g for 60 min, using 250 mL containers in an Avanti J‐26XPI centrifuge (Beckman Coulter, Brea, CA, USA) with a JLA 16.250 rotor. Supernatants were then ultracentrifuged at 135,000 × g for 105 min at 4°C in 25 mL bottles using an Optima L‐90K ultracentrifuge (Beckman Coulter) with a 50.2 Ti fixed rotor to pellet extracellular vesicles (EVs). Pellets were resuspended in 700 µL sterile PBS (pH 7.4) to obtain homogeneous suspensions, filtered through 0.45 µm Millex‐GP membranes (Merck Millipore, Burlington, MA, USA) to remove large particles, and stored at −80°C until use.

For further purification from co‐sedimented contaminants, PDNVs were processed by size‐exclusion chromatography (SEC) as previously described (Lopez de Las Hazas et al. 2023). Briefly, 30 mL SEC columns were packed with 20 mL Sepharose CL‐2B (Cat. number CL2B300, Sigma‐Aldrich, St. Louis, MO, USA) to form a matrix of 1.5 cm diameter × 12 cm height. Columns were preserved in 0.02% sodium azide in PBS at 4°C and equilibrated with 0.22 µm–filtered PBS (pH 7.4) before use. Filtered EV suspensions (700 µL) were loaded onto the columns, and 40 sequential fractions of 1 mL were collected by gravity elution with PBS. Columns were prepared specifically for each compound to avoid cross contamination. In addition, cleaning protocol were stablished by passing at least 3 volumes of PBS, 3 volumes of ethanol at 20%, and a restoring of 2 times of PBS.

### 2.3 Apoplast Wash Fluid Isolation

Apoplast wash fluid (AWF) was isolated from plant leaves using a modified infiltration–centrifugation method based on previously described protocols (O'Leary et al. 2014; Rutter and Innes 2016). Briefly, fresh leaves were harvested and thoroughly rinsed with distilled water to remove surface contaminants. Vacuum infiltration was performed by immersing leaves in MES infiltration buffer and applying repeated cycles of vacuum and atmospheric pressure until full infiltration was achieved (indicated by uniform darkening of the leaf tissue).

Infiltrated leaves were placed into 50 mL centrifuge tubes or a suitable holder within a swinging‐bucket rotor and centrifuged at 1000 × g for 10 min at 4°C to recover apoplastic wash fluid (AWF). The recovered fluid was collected and subjected to a second centrifugation step at 15,000 × g for 5 min at 4°C to remove residual cells, debris and particulate material. Later, EVs were isolated using UC conditions (135000 g during 105 min at 4°C).

### 2.4 Human Plasma EV Isolation

Plasma samples were obtained from healthy volunteers enrolled in a previously published hydroxytyrosol supplementation study (Del Saz‐Lara et al. 2025). Blood was collected in EDTA tubes, centrifuged at 1000 × g for 20 min at 4°C, and plasma was stored at −80°C until use. EVs were isolated following MISEV2018 guidelines (Thery et al. 2018) using sequential centrifugation, UC and purified by SEC. Briefly, 4 mL plasma was centrifuged at 5000 × g for 5 min and 10,000 × g for 15 min to remove cells and debris. The supernatant was ultracentrifuged at 100,000 × g for 105 min at 4°C (Beckman Coulter Optima L‐90K, rotor type), and the resulting pellet was resuspended in 500 µL sterile PBS (pH 7.4) and filtered through a 0.22 µm membrane to eliminate contaminants. SEC was subsequently performed by gravity elution with PBS, collecting 40 sequential fractions of 500 µL to remove other soluble co‐isolated proteins.

### 2.5 Bacterial Extracellular Vesicle (BEV) Isolation

*Pseudomonas aeruginosa* (strain PAO1) was cultured in Luria‐Bertani (LB) broth at 37°C with shaking until mid‐logarithmic phase. Cells were removed by centrifugation at 10,000 × g for 20 min. Supernatant was collected and centrifugation at 25,000 × g for 30 min to further remove other protein contaminants, and the supernatant was filtered through a 0.22 µm membrane. BMVs were pelleted by UC at 100,000 × g for 105 min at 4°C, washed with PBS, and the pellet resuspended in 0.7 mL of PBS. BEVs were further purified by SEC as described above.

### 2.6 Nanoparticle Tracking Analysis (NTA)

Particle size distribution, concentration and zeta potential of PDNVs, EVs and BMVs were determined using a ZetaView Twin instrument (Particle Metrix, Inning am Ammersee, Germany). Samples were diluted 1:10,000 in PBS for size and concentration measurements, and in Milli‐Q water for zeta potential analysis. Measurements were performed at 23–27°C on thawed samples. Each sample was analyzed in triplicate using identical detection settings.

### 2.7 Transmission Electron Microscopy (TEM)

PDNVs and BMVs morphology was examined by TEM using negative staining. Samples were applied to 200‐mesh copper grids for 3 min, stained with 2% uranyl acetate for 1 min, and air‐dried. Grids were imaged on a JEOL JEM‐1010 microscope operating at 100 kV at the Spanish National Centre for Electron Microscopy (CNME‐UCM, Madrid, Spain).

### 2.8 Protein Quantification

PDNV and BEV protein content was determined using the bicinchoninic acid (BCA) assay (Thermo Fisher Scientific, Madrid, Spain) according to the manufacturer's instructions. Bovine serum albumin (BSA) was used as a standard. Measurements were performed in triplicate for each sample.

### 2.9 ABTS Assay

ABTS radical cation (ABTS•^+^) was generated by mixing 7 mM ABTS in Milli‐Q water with 2.45 mM potassium persulfate and incubating in the dark at room temperature with gentle agitation for 12–16 h. The working solution was prepared by dilution in PBS (≈0.229 mM) to a final absorbance of 0.70 ± 0.02 at 734 nm. Reactions were performed in 96‐well plates by combining 175 µL ABTS•^+^ solution with 25 µL sample or Trolox standard. Controls contained 25 µL PBS or PBS with 1% SDS. For blanks, EVs in PBS at each working dilution was incorporated. Absorbance at 734 nm was recorded after 120 min. Trolox equivalent antioxidant capacity (TEAC) values were calculated relative to the Trolox calibration curve and expressed as mmol Trolox/L sample. Each sample was analyzed in duplicate from three independent preparations (*n* = 6).

### 2.10 ORAC Assay

The oxygen radical absorbance capacity (ORAC) assay was performed with adaptations from Davalos et al. (Davalos et al. 2004). Reactions were carried out in black 96‐well plates (Nunc MicroWell, Thermo Fisher Scientific, Madrid, Spain) at 37°C. Each well contained 150 µL fluorescein solution (800 nM in 0.075 M PBS, pH 7.4), 25 µL of sample or Trolox standard, and 25 µL of freshly prepared 156 mM AAPH solution in PBS. For blanks, PBS replaced the sample or AAPH as appropriate.

The reaction plate was shaken for 8 s prior to each reading. Fluorescence (excitation 485 nm, emission 520 nm) was recorded every minute for 100 min using a VICTOR Nivo Multimode Plate Reader (Perkin Elmer, Shelton, CT, USA). The area under the fluorescence decay curve (AUC) was calculated for samples, controls, blanks and Trolox standards. Net AUC for each sample was obtained by subtracting the corresponding blank value. TEAC was determined by interpolating the net AUC of the sample onto a standard curve generated from Trolox (25–100 µM) and expressed as µmol Trolox/L sample or µmol Trolox/g protein. Each sample was analysed at least in duplicate from three or more independent preparations.

#### 2.10.1 Analytical Sensitivity and Matrix Effects of the ORAC Assay

The limit of detection (LOD) and the limit of quantification (LOQ) were calculated based on the standard deviation (SD) of the response (σ) and the slope (S), using the equations 3.3 and 10σ/S, respectively, for each of the analyzed samples.

The potential matrix effect of EVs on the measurement of antioxidant activity was evaluated by spiking EV samples with a known concentration of hydroxytyrosol. To this end, the antioxidant activity of a 2.86 µg/mL hydroxytyrosol solution (25 µL added) and that of melon, blueberry and lemon EV samples (25 µL of sample) were analyzed separately. These results were compared with those obtained from samples containing 12.5 µL of a 5.72 µg/mL hydroxytyrosol solution and 12.5 µL of the corresponding EV sample, for which an additive antioxidant activity (predicted value) was expected. The matrix effect was determined as the difference between the experimentally measured antioxidant activity of the spiked sample and the predicted value, calculated as the sum of the individual antioxidant activities of hydroxytyrosol and the EV sample. A deviation of less than 10% was considered indicative of the absence of a significant matrix effect from the EVs.

### 2.11 Interlaboratory Validation

Interlaboratory validation was performed in two independent laboratories to assess the reproducibility and robustness of the ABTS and ORAC assays. Identical plant‐derived EV preparations were aliquoted and analyzed using harmonized protocols, including reagent preparation, assay conditions and normalization to protein content. The assays were conducted using different microplate readers—a VICTOR Nivo Multimode Plate Reader (PerkinElmer) in the originating laboratory and a FLUOstar Omega (BMG Labtech, Ortenberg, Germany) in the second laboratory—with instrument‐specific settings optimized locally while maintaining consistent wavelengths and temperature conditions (37°C for ORAC). Trolox calibration curves were independently generated in each laboratory.

Replicate‐level total‐protein‐normalized TEAC values were analyzed for plant‐derived EV preparations in two independent laboratories in three analytical blocks: ORAC‐EVs, ABTS‐EVs and ABTS‐EVs‐SDS. The analyzable dataset comprised six fruit matrices (strawberry, carrot, lemon, blueberry, ginger and melon). Tomato and onion were excluded a priori because they presented total protein concentrations below 0.01 mg/mL, which can generate artificially magnified protein‐normalized values and were therefore considered unreliable for quantitative interpretation. The statistical workflow used a cleaned long‐format dataset with one row per replicate and no imputation of missing values.

For each assay × fraction × fruit × laboratory combination, n, mean, SD and coefficient of variation (CV) were recalculated directly from the observed replicate values. Within‐laboratory repeatability was assessed descriptively using CV < 15%, consistent with commonly used bioanalytical validation practice (FDA. U. S.; Harmonisation., I. C.f 2022). Because a universal fixed interlaboratory CV threshold is not established and collaborative‐validation expectations are often concentration‐dependent (Horwitz and Albert 2006; International, A. 2016), cross‐laboratory performance was evaluated primarily by laboratory ratios, absolute and percentage differences, scatterplots and Bland–Altman analysis on log10‐transformed laboratory means (Bland and Altman 1986; Institute, C.a.L.S 2018). A CV < 20% criterion was retained only as a predefined pragmatic descriptor, interpreted alongside these complementary agreement measures. Pearson and Spearman correlations were also computed as descriptors of linear concordance and rank preservation, with greater interpretive emphasis placed on Spearman. An exploratory model restricted to ABTS data was additionally fitted to compare EVs and EVs‐SDS while adjusting for fruit and laboratory.

### 2.12 Loading of PDNVs With (Poly)Phenols

Resveratrol, gallic acid, quercetin and hydroxytyrosol were incorporated into filtered, precipitated EVs following established protocols (Ávila‐Gálvez et al. 2024). In brief, a concentrated solution of each compound was added to EVs (10% v/v). The mixture was subjected to passive incubation for one hour at room temperature in the dark under constant agitation (40 rpm). To remove unbound (poly)phenols and residual protein impurities, the loaded EVs were refined via SEC. Relevant EVs fractions were pooled and then concentrated through UC (100,000 g for 105 min). The resulting pellets were reconstituted in PBS, passed through a 0.22 µm sterile filter, and stored under aseptic conditions.

### 2.13 Oxidative Stress Assay

Intracellular oxidative stress was assessed using a reactive oxygen species (ROS)/Superoxide Detection Assay Kit (ab139476, Abcam, Cambridge, MA, USA), following the manufacturer's instructions. THP‐1 monocytes, EA.hy926 endothelial cells, and Caco‐2 intestinal epithelial cells were seeded in 12‐well plates and treated with plant‐derived PDNVs at 1, 2.5, 5, or 10% (v/v) for 24 h or at 4 h for EA.hy926. After treatment, cells were trypsinized, washed with assay buffer, and incubated with the oxidative stress detection solution for 30 min at 37°C in the dark. N‐acetyl‐L‐cysteine (NAC) and pyocyanin were included as negative and positive assay controls, respectively. Cells were acquired on a BD FACSCelesta SORP flow cytometer, and data were analysed using FlowJo software (Tree Star, Inc., Ashland, OR, USA). Intracellular ROS signal was quantified as mean fluorescence intensity (MFI) and expressed relative to untreated control cells (set to 100%).

### 2.14 Cell Viability Assay

THP‐1 monocytes, Caco‐2 enterocytes and EA.hy926 endothelial cells were maintained in DMEM or RPMI medium supplemented with 10 % FBS (Sigma Aldrich), 1 % L‐glutamine (Gibco, Thermo Scientific), and 1 % antibiotics (100 mg/mL penicillin and streptomycin) (Gibco) until reaching a confluence of 70%–80%. To evaluate the cytotoxicity of the EVs, cells were seeded in 96‐well plates at a density of 5 × 10^ (Xu et al. 2025) cells/cm^2^ and after 24 h, treated with EVs isolates at different concentrations (0%, 1%, 2%, 5%, 10% and 20%) for another 24 h. MTT (M2128, Sigma‐Aldrich) was added to each well at a final concentration of 0.5 mg/mL for 2 h. After MTT removal, DMSO was added to dissolve MTT‐formazan crystals, and absorbance measured at 570 nm. Data are shown as the mean of at least three independent experiments, each performed in technical triplicate.

### 2.15 Western Blot

To validate the success of the isolation procedure, the presence of EV‑specific marker proteins was assessed by Western blotting. Protein samples (≈50 µg of protein per sample) were denatured under reducing conditions and resolved on either 10% or 7.5% sodium dodecyl sulfate‑polyacrylamide/bisacrylamide gels. Following electrophoresis, proteins were transferred onto 0.22‑µm nitrocellulose membranes (Millipore) and blocked with 5% skimmed milk (Bio‑Rad) for 30 min at room temperature. Membranes were then incubated overnight at 4°C with the appropriate primary antibodies. After primary incubation, the membranes were probed with secondary anti‑rabbit or anti‑mouse antibodies conjugated to Alexa Fluor 680 or IRDye 800 for 45 min at room temperature. Each incubation step was followed by three washes with TBST buffer, and the blots were finally visualized using an Odyssey infrared imaging system (LI‑COR).

### 2.16 Statistical Analysis

Data are presented as mean ± SD. Normality was assessed using the Shapiro–Wilk test. Comparisons among groups were performed using one‐way ANOVA followed by Tukey's post hoc test, Student's *t*‐test where appropriate, or two‐way ANOVA followed by Tukey's or Dunnett's post hoc test for multi‐factor experiments, as indicated in the corresponding figure legends (GraphPad Prism v9.3.1). Correlations were assessed by Pearson's coefficient. A *p*‐value < 0.05 was considered statistically significant.

## 3 Results

### 3.1 Development of Microplate‐Based Antioxidant Assays Adapted to PDNVs and EVs

To enable reliable quantification of antioxidant activity in nanovesicles and EVs, we first adapted and optimized ABTS, DPPH and ORAC assays for low‐input, highly purified EV samples, avoiding co‐isolation bias, that could generate a standardized antioxidant assay specific for EV samples.

#### 3.1.1 Optimization of the ABTS Radical Assay for Measuring Antioxidant Activity of Isolated PDN‐Vs

To assess the antioxidant activity of isolated PDNVs, we adapted the ABTS radical cation decolorization assay originally developed by Re et al. (Re et al. 1999) into a microplate‐compatible format. In the original protocol, the ABTS radical solution in PBS was adjusted to an absorbance of 0.70 ± 0.02 at 734 nm, with 990 µL of ABTS working solution mixed with 10 µL of sample in a total reaction volume of 1 mL.

For high‐throughput screening, the assay was miniaturized to a final reaction volume of 200 µL using a 96‐well flat‐bottom microplate. The scaled‐down reaction used 198 µL of ABTS radical solution and 2 µL of sample. To determine the ABTS concentration required to maintain an absorbance of 0.70 ± 0.02 in this miniaturized format, a standard curve was generated (Figure S1A), identifying 0.2 mM as the optimal concentration.

Given the expected low antioxidant activity of the target PDNVs—obtained via UC and further purified by size exclusion chromatography (SEC)—the assay was further optimized for increased sample volumes (5 and 25 µL), while maintaining the total reaction volume at 200 µL. Using the ABTS standard curve, the concentrations required to maintain an equivalent radical load in 195 and 175 µL of ABTS solution were calculated as 0.205 and 0.229 mM, respectively. Appropriate blanks (PDNVs in PBS) were used in every determination.

Trolox standard curves were generated for each assay condition (sample volumes of 2, 5 and 25 µL), yielding IC_50_ values of 2.709, 1.132 and 0.227 mM, respectively (Figure 1A–C). Method validation was performed using three phenolic compounds and EVs isolated from broccoli inflorescences as a reference sample. All three assay configurations produced consistent results, with a CV < 10% (Figure 1D).

**FIGURE 1:**
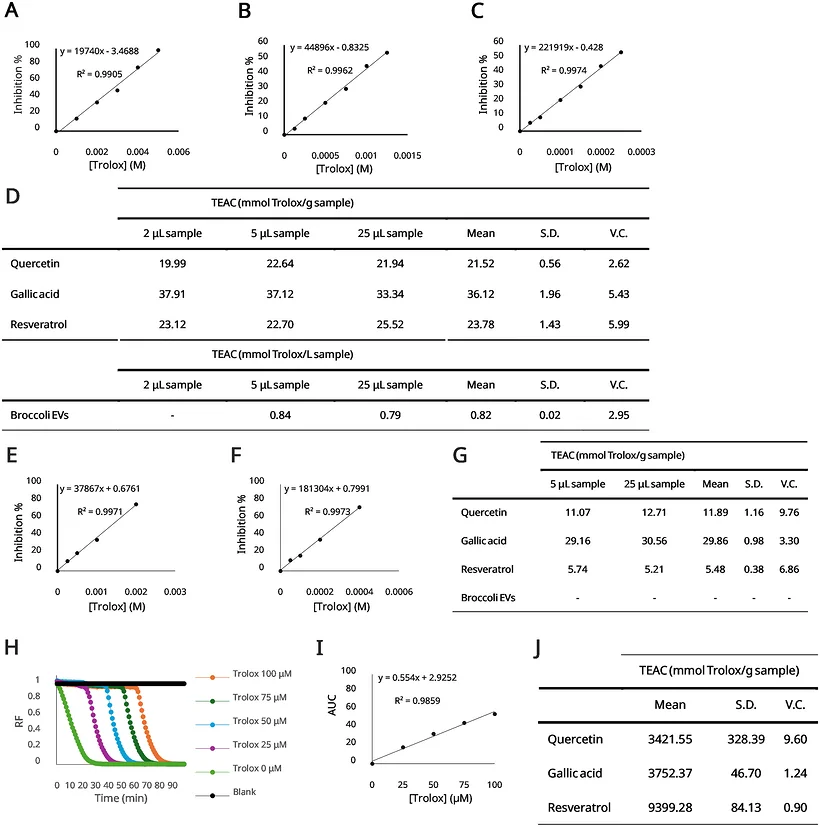
Optimization and validation of antioxidant assays (ABTS, DPPH, ORAC) for PDNVs analysis. (A–C) Trolox standard curves for ABTS assay radical inhibition (%) with 2 (A), 5 (B), or 25 µL (C) showing IC_50_ values of 2.709, 1.132 and 0.227 mM, respectively. (D) Validation of ABTS assay using three phenolic reference compounds and broccoli‐derived EVs, demonstrating a CV <10%. (E, F). Trolox standard curves for DPPH assay radical inhibition (%) with 5 µL (E) and 25 µL (F), with IC_50_ values of 1.334 and 0.323 mM, respectively. (G) Validation of DPPH assay using phenolic reference compounds. (H) Fluorescence decay curves for Trolox standards (25–100 µM) in ORAC assay. (I) Standard curve of net area under the curve (AUC) versus Trolox concentration. (J) Validation of ORAC assay using phenolic reference compounds. Data are presented as mean ± SD (*n* ≥ 3).

However, it is important to note that the 2 µL sample condition was insufficient to detect antioxidant activity in broccoli‐derived NVs, as the observed radical inhibition did not exceed 20%, likely due to their low antioxidant potential.

#### 3.1.2 Optimization of the DPPH Radical Assay for Antioxidant Activity Assessment of Purified PDNVs

The DPPH radical scavenging assay was similarly adapted for microplate‐based analysis to evaluate the antioxidant activity of EVs. The protocol was derived from the original method described by Brand‐Williams et al. (Brand‐Williams et al. 1995), where 975 µL of DPPH radical solution (adjusted to 0.60 absorbance units) was mixed with 25 µL of sample, resulting in a total volume of 1 mL.

To miniaturize the assay for a 96‐well format, the method was scaled down to a final reaction volume of 200 µL. Two sample volumes were tested: (i) 5 µL of sample with 195 µL of DPPH solution, and (ii) 25 µL of sample with 175 µL of DPPH solution. The DPPH concentrations required to achieve an initial absorbance of 0.60 at 517 nm were determined using a DPPH standard curve (Figure S1B), corresponding to 0.160 mM for the 195 µL condition and 0.200 mM for the 175 µL condition (both prepared in methanol).

Trolox standard curves, prepared using Trolox dissolved in PBS (1X), were generated for both sample volumes. The calculated IC_50_ values were 1.334 mM for the 5 µL condition and 0.323 mM for the 25 µL condition (Figure 1E,F). Method validation was confirmed by assessing the antioxidant activity of three phenolic reference compounds, yielding consistent results with a CV below 10% (Figure 1G).

However, this method proved unsuitable for evaluating the antioxidant capacity of EVs isolated from broccoli inflorescences (via UC and SEC). No measurable radical inhibition was observed. In contrast, an increase in absorbance beyond the initial 0.60 units was detected, likely due to a combination of low antioxidant activity and sample‐specific optical interference, as confirmed by elevated absorbance in the sample blanks.

#### 3.1.3 Optimization of the ORAC Assay for Measuring Antioxidant Activity of Isolated PDNVs

The ORAC assay was optimized based on our previous work (Nieto et al. 2024), with modifications from the method originally described by Davalos et al. (Davalos et al. 2004). A microplate‐based protocol with a total reaction volume of 200 µL was established, comprising 150 µL of fluorescein solution, 25 µL of AAPH (2,2'‐azobis(2‐methylpropionamidine) dihydrochloride) solution (or PBS for blanks), and 25 µL of sample or Trolox (or PBS for controls and blanks). Fluorescence intensity was recorded every minute for 100 min.

The fluorescein concentration was optimized at 800 nM in PBS (0.075 M, pH 7.4), as this was the maximum concentration that maintained linear fluorescence response (Figure S2B). Excitation/emission wavelengths were set at 480/530 nm, which provided stronger fluorescence signals compared to the alternative 460/530 nm setup (Figure S2A,B), which also works well.

To determine optimal oxidative stress conditions, two AAPH concentrations (76.5 and 153 mM) were freshly prepared in PBS and assayed at two temperature conditions (20°C or 37°C). The 153 mM AAPH solution, prepared in PBS at 37°C, was selected based on its ability to reduce fluorescein intensity to near‐baseline levels within approximately 20 min (i.e., 20% of total experiment time), thereby preserving a wide dynamic range for antioxidant detection. In contrast, the lower concentration extended fluorescein decay to 40% of the experiment duration, narrowing the detection window (Figure S2C).

The Trolox standard curve was established to ensure full depletion of antioxidant activity within the 100‐min assay duration. Trolox concentrations of 25, 50, 75 and 100 µM were evaluated (Figure 1H), and the corresponding net area under the curve (AUC) was calculated to generate the standard curve (Figure 1I).

Method validation was carried out using the three previously assessed phenolic compounds (Figure 1J) and EVs isolated from broccoli inflorescences (Figure S2D), confirming reliable measurement of radical scavenging activity. Notably, while EVs exhibited antioxidant capacity, a sharp initial drop in fluorescence was observed upon their addition to the fluorescein solution (Figure S2D). This effect, unrelated to radical scavenging, led to artificially low initial fluorescence readings (approximately 20% below the expected baseline), potentially underestimating antioxidant activity if calculated relative to the PBS blank.

To address this, relative fluorescence values for EVs were normalized against a dedicated *self‐blank* control consisting of 150 µL fluorescein solution, 25 µL PBS, and 25 µL of EV sample (Figure S2D). When EV antioxidant capacity was calculated using the PBS blank, it was underestimated (169.11 ± 1.20 µM Trolox/L). In contrast, referencing the *self‐blank* yielded a higher and more accurate value (195.32 ± 1.29 µM Trolox/L), highlighting the importance of sample‐specific baseline correction for EV analysis.

Analytical validation of the optimized assay, including determination of LoD, LoQ and matrix effects in representative PDNV preparations, is presented in Section 3.3.

### 3.2 Optimization of PDNV Disruption Protocols for Assessing Total Antioxidant Activity

The antioxidant mechanisms associated with PDNVs remain largely unresolved, particularly in terms of whether their bioactivity arises from surface components or encapsulated cargo. To address this, we developed and evaluated several PDNV disruption protocols to investigate the contribution of vesicle cargo to antioxidant activity (Figure 2). The central aim was to differentiate between the antioxidant activity attributable solely to the EVs membrane and that derived also from their full biochemical content upon membrane disruption.

**FIGURE 2:**
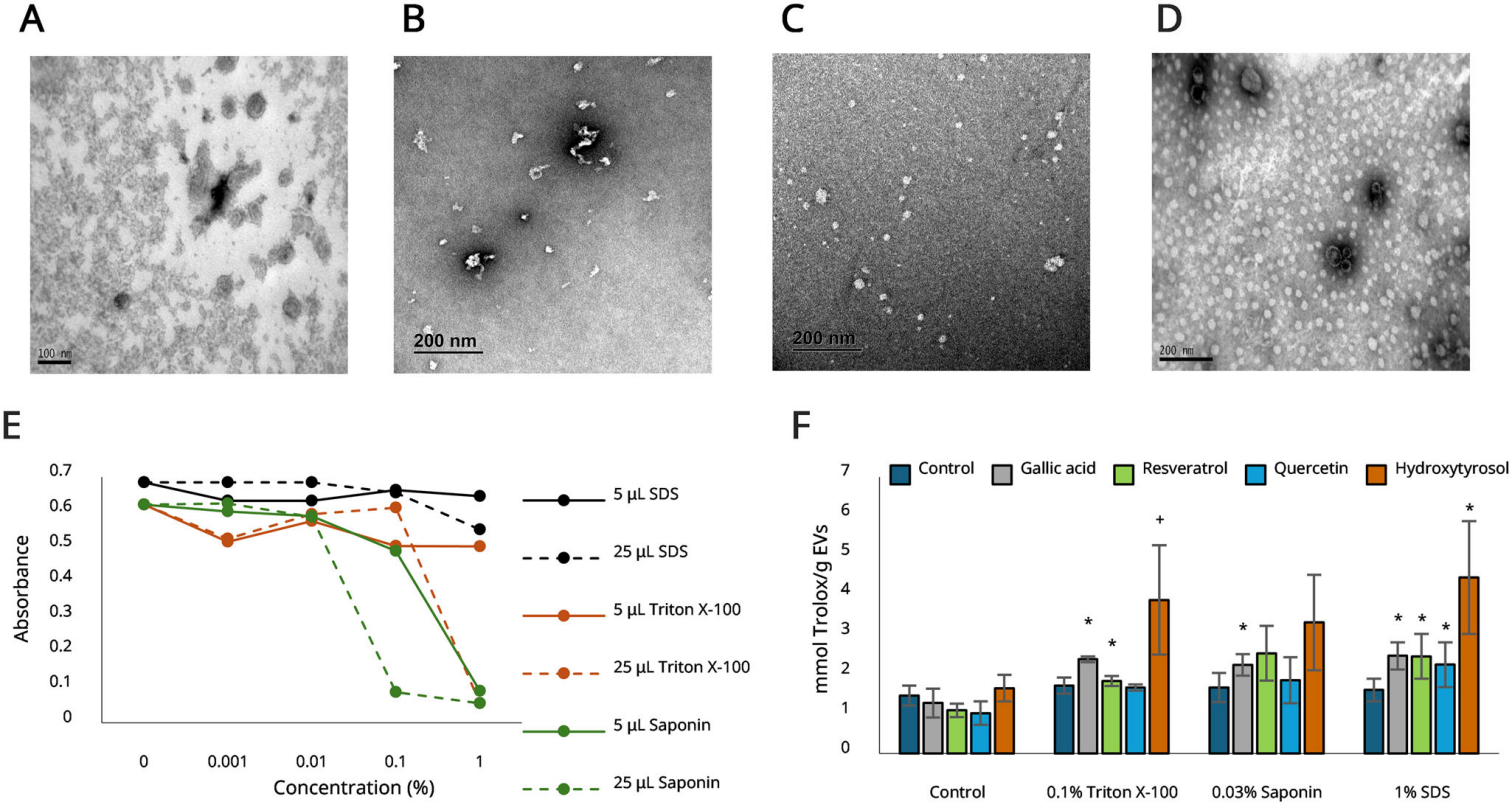
Vesicle integrity modulates vesicles antioxidant activity. (A–D) TEM of broccoli inflorescence EVs left untreated (A) or disrupted with 1% SDS (B), 0.1% Triton X‐100 (C), or 0.03% saponin (D). Scale bars represent 200 nm. (E) Effect of increasing detergent concentrations on ABTS radical absorbance at two EV input volumes (5 or 25 µL). (F) Antioxidant activity of native or phenolic‐loaded EVs (gallic acid, resveratrol, quercetin, or hydroxytyrosol) measured by ABTS after treatment with SDS, Triton X‐100, or saponin. Antioxidant activity is expressed as mmol Trolox equivalents per gram of EV protein. Data are shown as mean ± SD (*n* = 4). *p ≤ 0.05 versus the respective untreated control (Tukey's post hoc test).

We hypothesized that treatment‐induced disruption of PDNV membranes would release their internal cargo, potentially enhancing measurable antioxidant activity. Conversely, intact EVs would primarily reflect antioxidant contributions from surface proteins and lipids, limiting observable activity. Additionally, the possibility of solvent and efflux interactions affecting antioxidant measurements was also considered.

Three chemical disruption agents were pre‐selected based on their known ability to permeabilize or lyse membrane structures: sodium dodecyl sulfate (SDS), Triton X‐100, and saponin. Prior to protocol optimization, the compatibility of each agent with the ABTS and ORAC antioxidant assays was evaluated to exclude potential assay interferences (Figure 2E and Figure S3).

#### 3.2.1 Compatibility of Disruption Agents With ABTS and ORAC Assays

In the ABTS assay, SDS was compatible at concentrations up to 1% (v/v), showing minimal interference (Figure 2E). Triton X‐100 exhibited significant assay interference, necessitating a maximum concentration of 0.1% when using 25 µL of sample. Saponin was tolerated at concentrations up to 0.01%. Based on these findings, the working concentrations for further analyses were set at 1% SDS, 0.1% Triton X‐100 and 0.03% saponin.

To confirm assay compatibility and validate treatment conditions, we prepared two PDNV sample types from broccoli inflorescences: (i) Naturally occurring EVs (native EVs) and (ii) EVs loaded with selected phenolic compounds (gallic acid, resveratrol, quercetin or hydroxytyrosol). Both types were isolated via ultracentrifugation followed by size‐exclusion chromatography (UC + SEC). Native PDNVs were then either left untreated as control or subjected to disruption using the above‐validated concentrations of SDS, Triton X‐100, or saponin. For treatment, concentrated solutions of each agent were added to the EV samples to achieve the desired final concentrations, followed by vigorous mixing for 1 min. Serial dilutions were performed using PBS buffers containing the respective disruption agent to maintain treatment consistency. Corresponding buffer‐only controls and treatment disruption‐agent (SDS, Triton X‐100 or saponin) controls were also included.

#### 3.2.2 Disruption Unmasks Cargo‐Derived Activity in Loaded PDNVs

Antioxidant activity was quantified using the ABTS assay (25 µL protocol), and results were normalized to PDNV protein content, expressed as mmol Trolox equivalents per gram of PDNV protein (mmol TE/g protein). Protein content was selected as a practical normalization factor to express antioxidant activity relative to vesicle abundance, providing a consistent metric across samples despite differences in cargo composition. As shown in Figure 2F, native PDNVs and untreated phenolic‐loaded PDNVs displayed similar ABTS antioxidant activity, consistent with a predominant contribution of membrane‐associated components under intact conditions. In contrast, phenolic‐loaded PDNVs showed higher antioxidant activity after treatment with SDS, Triton X‐100, or saponin than their respective untreated controls, indicating that membrane disruption can unmask cargo‐associated antioxidant activity. While a significant increase in antioxidant activity after membrane disruption was observed for most phenolic‐loaded PDNVs (*p* ≤ 0.05), the magnitude of this increase was greatest in hydroxytyrosol‐loaded PDNVs, consistent with their higher loading efficiency (Figure S4). These results primarily serve as a proof‐of‐concept to validate that the disruption protocol can successfully unmask encapsulated antioxidant activity. The variation in loading reinforces the necessity of using such disruption methods to capture the full biochemical potential of PDNVs, particularly when cargo concentrations are low or vary significantly by compound type. So, these findings confirm that the disruption protocols effectively release antioxidant‐active cargo and that the ABTS assay does not fully capture total antioxidant potential unless PDNV membranes are compromised.

#### 3.2.3 Disruption Agents Are Incompatible With ORAC Assay

The potential interferences of SDS, Triton X‐100, and saponin with the ORAC assay were similarly evaluated, focusing on their effects on fluorescence intensity (fluorescein degradation) and AAPH radical activity (Figure S3). All three agents caused concentration‐dependent quenching of fluorescence, with saponin exhibiting unacceptable levels of interference across all tested concentrations. While low concentrations (0.01%) of SDS and Triton X‐100 were less disruptive to fluorescence, additional issues were observed upon interaction with AAPH.

Specifically, both Triton X‐100 and saponin substantially altered AAPH‐derived fluorescence kinetics, making them unsuitable for inclusion in the ORAC protocol. Although SDS showed relatively lower interference at very low concentrations (≤0.001%), treatment‐dependent, nonspecific effects on AAPH activity were observed across different EV concentrations, raising concerns about assay reliability and reproducibility.

Taken together, these results indicate that none of the tested chemical disruption protocols (SDS, Triton X‐100, or saponin) are compatible with the ORAC assay, primarily due to their interfering effects on key assay components (fluorescein and AAPH). Thus, these agents should not be employed in conjunction with ORAC for evaluating the total antioxidant capacity of PDNV samples.

### 3.3 Application to PDNVs and EVs

#### 3.3.1 Antioxidant Activity Decreases Dramatically Upon Purification

Following the optimization of the ABTS and ORAC antioxidant assays, and the development of an effective EVs disruption protocol for assessing total antioxidant capacity via the ABTS method, we evaluated PDNVs isolated from various plant sources. Isolation was performed by a dual‐step isolation protocol (UC + SEC) designed to remove co‐isolated soluble proteins and contaminants. This approach follows the MISEV2018 and MISEV2023 guidelines (Thery et al. 2018; Welsh et al. 2024), which recommend using two consecutive methods to achieve highly purified EVs. Isolated PDNVs from different plant sources (Figure 3) were compared their antioxidant activity between the different step of isolation. For comparative analysis, three sample types were assessed for each plant: (i) the initial extract (post 35,000 × g centrifugation), (ii) UC pellet and (iii) PDNVs purified by UC followed by SEC (Figure 4).

**FIGURE 3:**
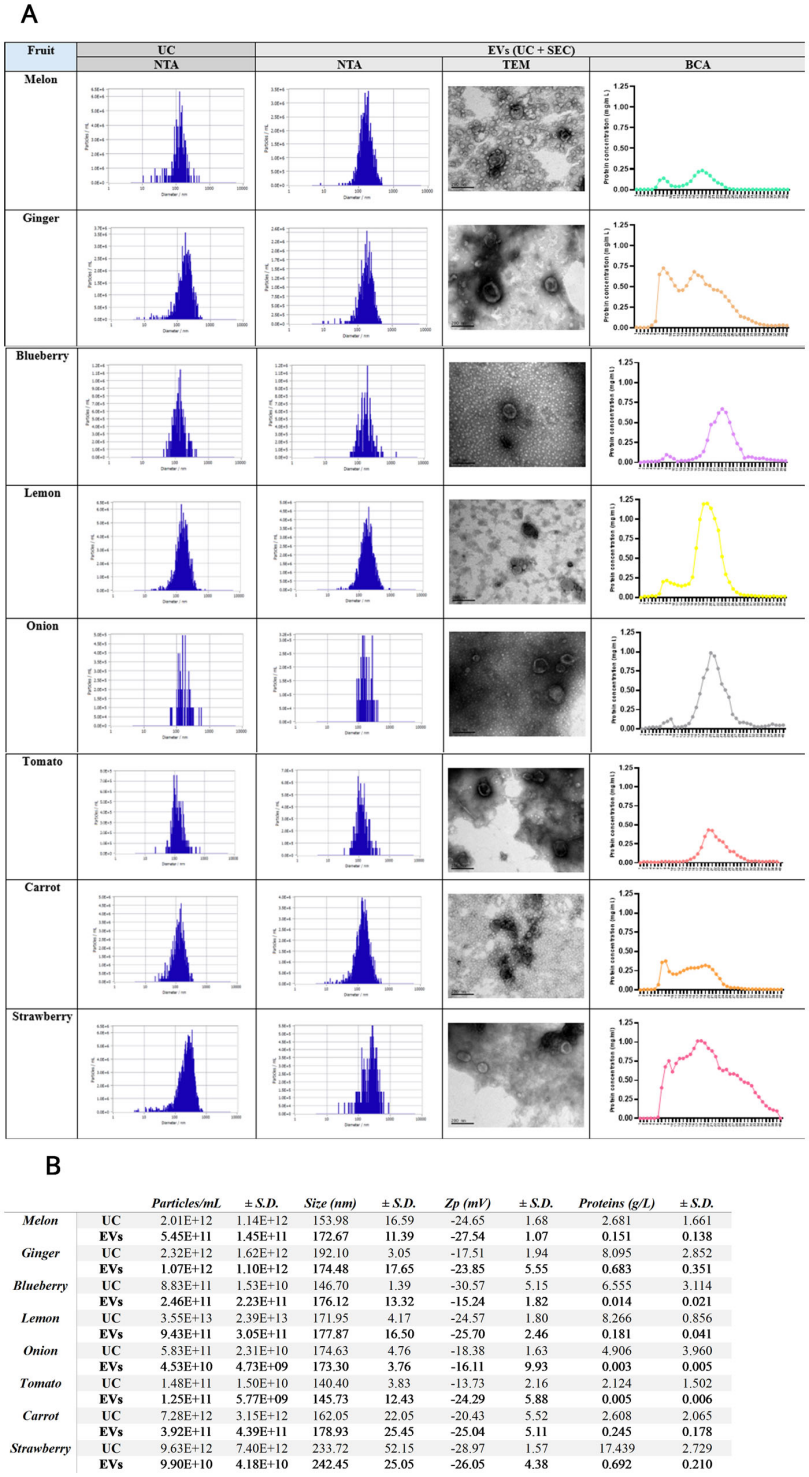
Characterization of fruit‐ and vegetable‐derived nanovesicles isolated by UC or UC combined with size‐exclusion chromatography (UC + SEC). (A) Representative NTA, size distribution profiles, TEM images (scale bars: 200 nm) and protein elution profiles for EVs isolated by UC (left) or UC + SEC (right). (B), Summary of particle concentration (particles/mL), mean diameter (nm), zeta potential (mV) and protein concentration (g/L sample) for UC and UC + SEC preparations (*n* = 3, mean ± SD). EVs, extracellular vesicles; UC, ultracentrifugation; SEC, size‐exclusion chromatography; NTA, nanoparticle tracking analysis; TEM, transmission electron microscopy; BCA, bicinchoninic acid assay.

**FIGURE 4:**
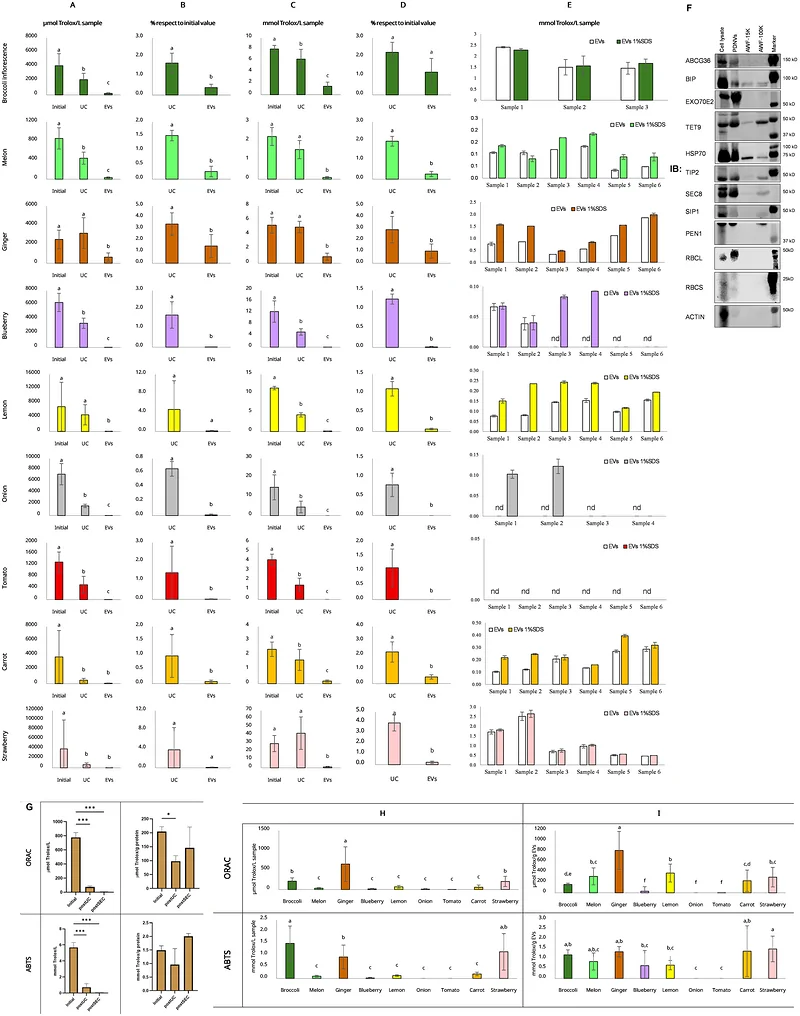
Antioxidant activity of fruit‐ and vegetable‐derived EVs isolated by UC or UC + SEC. (A, B) Antioxidant activity measured by ORAC across isolated fractions, expressed as µmol Trolox/L sample (A) and as percentage of activity retained in UC and EV fractions relative to the initial sample (B). (C–E), Antioxidant activity measured by ABTS across isolation fractions (mmol Trolox/L sample) (C), retained activity relative to initial isolated (D), and effect of 1% SDS disruption compared with untreated EVs (E). (F) Western blot analysis of *Nicotiana benthamiana* AWF. (G) Antioxidant activity measured by ABTS and ORAC in AWF from *Nicotiana benthamiana* leaves. (H–I), Antioxidant activity of UC + SEC EV preparations quantified by ORAC (µmol Trolox/L sample) and ABTS (mmol Trolox/L sample) (H), and normalized to protein content (µmol Trolox/g protein) for ORAC and ABTS (mmol Trolox/g protein) (I). ^a,b,c^ indicate significant differences among the samples through Tukey post‐hoc test (*p* ≤ 0.05). * *p* ≤ 0.05 versus untreated EVs (Tukey post‐hoc test). (*n* = 6). Statistical differences in panel G were assessed by two‐way ANOVA, followed by Tukey's post‐hoc test for multiple comparisons. *p < 0.05; ***p < 0.001.

*ORAC Analysis of Antioxidant Activity*. The ORAC assay revealed a marked reduction in antioxidant activity in both UC and purified EV fractions compared to the initial extracts (Figure 4A). When values were corrected for sample dilution relative to the initial extract (Figure 4B), this trend remained consistent. UC led to a ≥90% decrease in antioxidant activity, with most samples showing <5% of the original TEAC. Purified EVs showed even lower activity, often <1% of the initial value—except for ginger‐derived nanovesicles, which retained comparatively higher antioxidant activity.

*ABTS Analysis and Impact of PDNV Disruption*. A similar pattern was observed with the ABTS assay: antioxidant activity decreased significantly following UC and subsequent SEC purification (Figure 4C). After correcting for sample concentration (Figure 4D), both UC and purified EV fractions retained minimal antioxidant capacity. However, upon EV disruption using 1% SDS, several purified PDNV samples demonstrated increased antioxidant activity (Figure 4E), suggesting that cargo components also contribute to the total potential antioxidant activity. Notably, no increase was observed in most of PDNV samples derived from blueberry, broccoli inflorescence, onion, tomato, carrot and strawberry.

Overall, a substantial reduction (>90%) in antioxidant activity was observed from the initial extracts to UC pellets and further to SEC‐purified PDNVs, as measured by both ORAC and ABTS. Even after correcting for dilution, purified PDNV fractions retained only a small fraction of the initial antioxidant capacity. These results indicate that most antioxidant activity in crude extracts arises from non‐vesicular components.

#### 3.3.2 Apoplastic Fluids Confirm Low Antioxidant Activity of Purified EVs

To further validate that the measured antioxidant activity in plant‐derived EV preparations is largely driven by non‐vesicular components, we analyzed EVs isolated from AWF obtained from *Nicotiana benthamiana* leaves using an infiltration–centrifugation approach. This method minimizes mechanical disruption and is considered to better reflect native extracellular vesicle populations.

To distinguish PDNVs isolated from homogenized tissue from bona fide extracellular vesicles isolated from the apoplast, we compared the protein composition of total cell lysates, PDNVs, AWF after 15,000 × g centrifugation, and AWF‐derived vesicles recovered by UC (Figure 4F). The canonical EV markers SYP121 (PEN1) and HSP70 were enriched in the AWF‐derived vesicle fraction, while TET9, TIP2 and SEC8 displayed similar enrichment patterns. In contrast, ABCG36 (PDR8/PEN3) was abundant in total cell lysates and PDNVs but was essentially absent from the AWF‐derived vesicle fraction, indicating that this plasma membrane transporter is associated with homogenate‐derived vesicles rather than apoplastic extracellular vesicles. A similar pattern was observed for EXO70E2. Finally, ACTIN and RBCS1A were detected almost exclusively in total cell lysates and were absent or nearly undetectable in the extracellular fractions, supporting reduced contamination by cytosolic and chloroplastic proteins.

Antioxidant activity was assessed at three stages: (i) AWF following 15,000 × g centrifugation, (ii) UC pellets and (iii) EVs purified by UC followed by SEC. As observed for fruit‐derived samples, antioxidant activity decreased progressively with each purification step (Figure 4G), with very low activity detected in the SEC‐purified EV fraction. These results are consistent with those obtained from mechanically derived plant extracts and further support the conclusion that most of the measured activity appears to originate from soluble non‐vesicular components, although partial removal of physiologically associated EV corona components during purification cannot be excluded. Importantly, the low antioxidant activity observed in highly purified AWF‐derived EVs reinforces that bona fide plant EVs possess limited intrinsic antioxidant capacity under basal conditions.

#### 3.3.3 Analytical Sensitivity and Matrix Effects of the ORAC Assay in PDNV Preparations

Because purified PDNVs exhibited very low antioxidant activity after UC and SEC purification, we next evaluated the analytical sensitivity of the ORAC assay within representative PDNV matrices. Limits of detection (LoD) and quantification (LoQ) were determined independently in two laboratories using different fluorescence plate readers (FLUOstar Omega and NIVO) (Figure S5). The assay exhibited matrix‐dependent analytical sensitivity. Lemon‐ and blueberry‐derived PDNVs showed LoD values ranging from approximately 0.003–0.013 g/L protein and LoQ values from approximately 0.008–0.040 g/L protein, whereas melon‐derived PDNVs exhibited higher limits (LoD ≈ 0.12 g/L protein; LoQ ≈ 0.40 g/L protein), indicating lower analytical sensitivity in this matrix.

To investigate whether PDNVs interfere with ORAC measurements, spike‐in recovery experiments were performed using hydroxytyrosol (HT) as a reference antioxidant (Figure S6). The experimentally observed antioxidant activities closely matched the expected additive values in both independent laboratories. Percentage deviations remained below 10% for all PDNV preparations, with recoveries ranging from approximately 91%–98% in Laboratory 1 and 95%–100% in Laboratory 2. These results indicate negligible PDNV‐driven matrix effects and demonstrate that the vesicle matrix neither substantially quenches nor enhances ORAC measurements under the experimental conditions employed.

Collectively, these experiments demonstrate that the optimized ORAC assay possesses sufficient analytical sensitivity to quantify the low antioxidant activities observed in purified PDNV preparations while exhibiting minimal interference from the vesicle matrix.

#### 3.3.4 Influence of Botanical Origin

Antioxidant activity differed across botanical sources (Figure 4H,I). Ginger‐, strawberry‐ and broccoli‐inflorescence‐derived EVs showed the highest raw antioxidant activity across the ORAC and ABTS assays (Figure 4H). Normalization to PDNV protein content revealed a similar overall pattern while reducing variability related to PDNV yield, with ginger nanovesicles remaining the most antioxidant‐active preparations (Figure 4I). Differences among botanical sources were less pronounced in the ABTS protein‐normalized analysis.

Protein quantification revealed significant variability in PDNV yield among plant sources (Figure 3B). UC fractions contained higher protein content than SEC‐purified nanovesicles, likely due to co‐isolation of non‐EV contaminants. Consequently, the TEAC/BCA ratio was consistently lower in UC samples (data not shown), indicating overestimation of antioxidant activity due to contaminant interference. These findings support the use of protein normalization as a practical proxy for PDNV abundance in purified samples.

#### 3.3.5 Protein Normalization Enables Cross‐Sample Comparison

Weak correlation was observed between antioxidant activity (ORAC) and protein content in isolated PDNVs (*r* ^2^ = 0.2851) (Figure 5A). However, ginger nanovesicles represented a clear outlier. When ginger data were excluded, the correlation improved substantially (*r* ^2^ = 0.7877), suggesting a proportional relationship between PDNV concentration and antioxidant capacity (Figure 5B).

**FIGURE 5:**
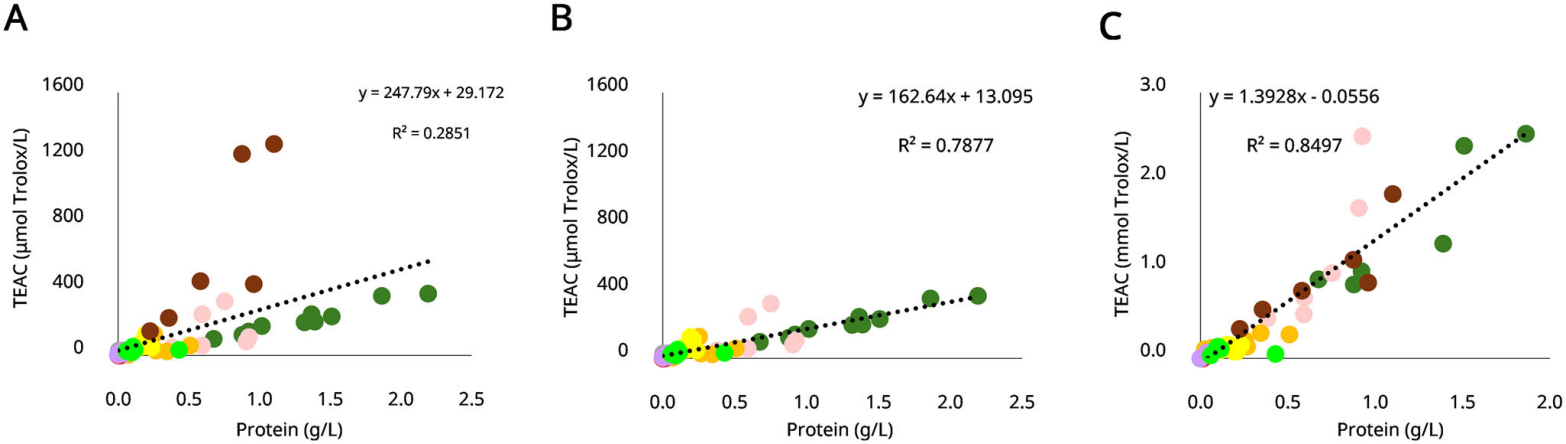
Correlation between PDNVs antioxidant activity and protein content. (A) Correlation between antioxidant activity measured by ORAC and EV protein content, (B) correlation excluding ginger‐derived EVs. (C) Correlation between antioxidant activity measured by ABTS assay and EV protein content. TEAC, TEAC.

For the ABTS assay, a strong correlation was found between TEAC values and protein content (*r* ^2^ = 0.8497), even without excluding any outliers (Figure 5C), further supporting protein content as a reliable proxy for PDNV abundance in evaluating antioxidant capacity.

To account for the possibility that protein content may vary across vesicles from different sources (mammalian, plant and bacterial), we also performed correlation analyses using NTA particle concentration as an alternative normalization metric. In contrast to the strong correlations observed with protein content, only weak relationships were found between antioxidant activity and particle concentration, both for ORAC (*r* ^2^ = 0.2001; Figure S7B) and ABTS (*r* ^2^ = 0.0603; Figure S7C).

#### 3.3.6 Isolation Method Influences Antioxidant Activity

Exploratory comparison of different isolation methods (UC, PEG precipitation and TFF) suggested that the purification strategy can influence the measured antioxidant activity, as illustrated for lemon and ginger EVs (Figure 6). Antioxidant activity in the initial extract supernatant (post 35,000 × g centrifugation) and in UC pellets prior to SEC purification appeared broadly comparable across methods, whereas differences became more evident after purification, particularly in the ORAC assay. Consistent with previous observations, antioxidant activity decreased markedly upon purification, supporting a substantial contribution of co‐isolated components in less refined preparations.

**FIGURE 6:**
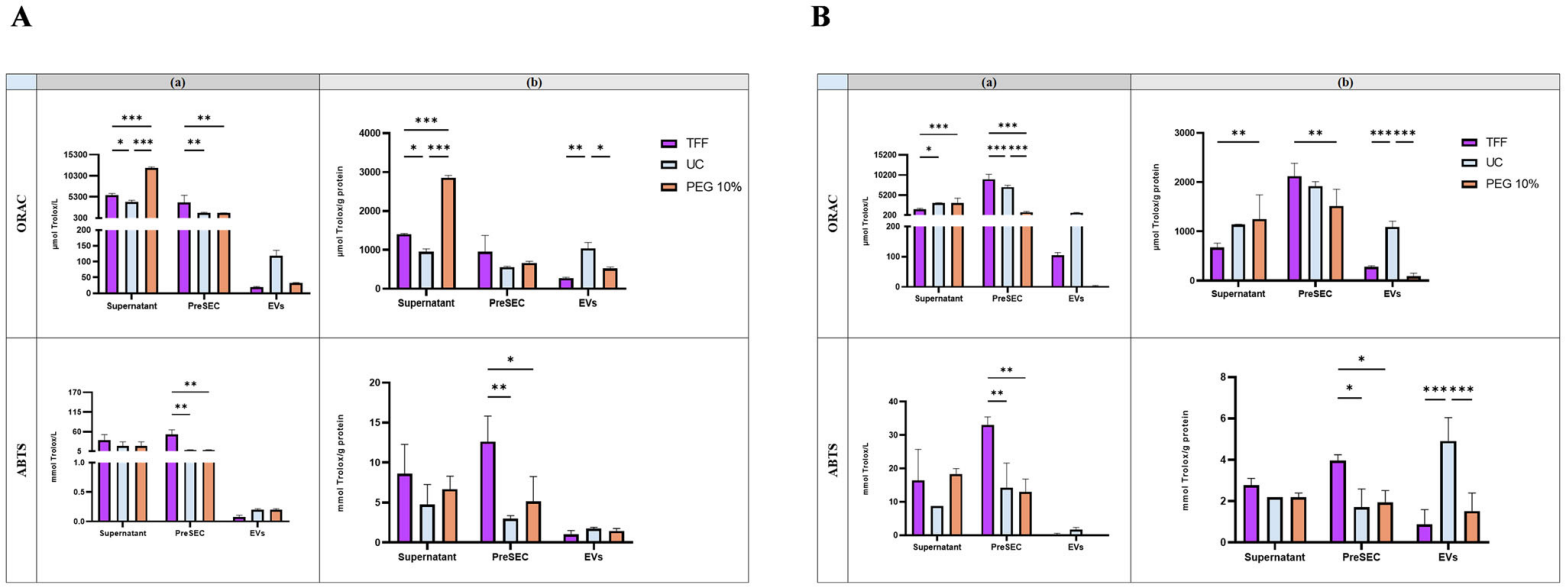
Effect of isolation method on PDNVs antioxidant activity. (A, B) Antioxidant activity of EVs isolated from lemon (A) and ginger (B) using tangential flow filtration (violet, TFF), ultracentrifugation (blue, UC), or 10% polyethylene glycol precipitation (orange, PEG 10%). Total antioxidant activity (left) and antioxidant activity normalized to EV protein content (right) were assessed by ABTS and ORAC assays. Data are shown as mean ± SD (*n* = 2 independent preparations). Owing to the limited sample size, these data should be interpreted as exploratory and descriptive.

#### 3.3.7 Co‐Isolate Contaminants Retain a High Antioxidant Activity

To determine the origin of the antioxidant activity detected in supernatants and pre‐SEC samples, we profiled antioxidant activity across all 40 fractions obtained by SEC. We first assessed whether different isolation methodologies influenced the co‐isolation of contaminating components and their distribution across fractions. Protein content, used as a proxy for contaminants (BCA assay), revealed that TFF resulted in higher levels of protein co‐isolates, particularly in fractions F17–F21 for lemon nanovesicles (Figure 7A) and F15–F22 for ginger EVs (Figure 7A). In contrast, UC yielded a more defined PDNV peak (fractions F6–F8), characterized by higher particle numbers and protein concentration, indicative of enriched nanovesicle fractions (Figure 7B).

**FIGURE 7:**
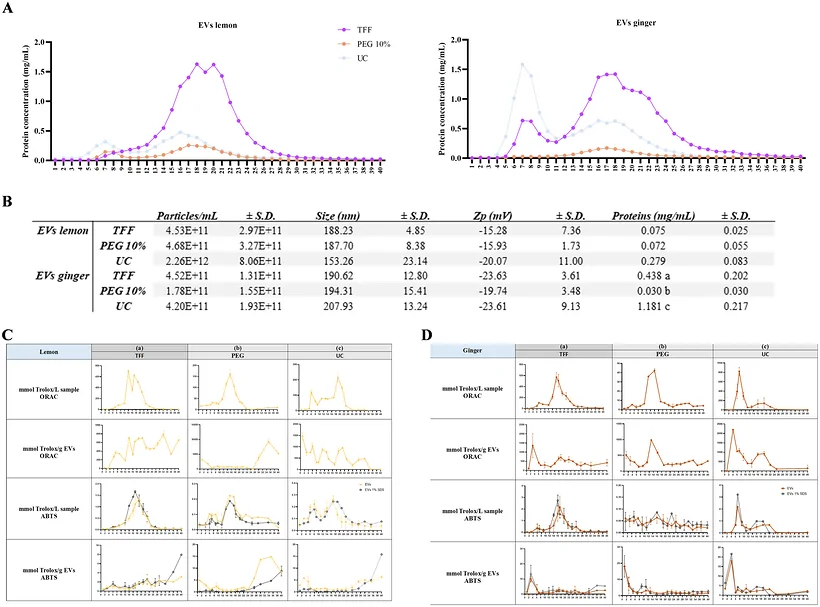
Distribution of protein contaminants and antioxidant activity across SEC fractions reveals enrichment of non‐vesicular antioxidant capacity (A) Protein concentration profiles (BCA assay) of 40 fractions collected after SEC purification of lemon and ginger EVs obtained using different isolation methods. (B) Characterization of EV‐enriched fractions, including particle concentration (particles/mL), size (nm) and ζ‐potential (mV) determined by NTA, along with protein concentration (mg/mL) measured by BCA. Data are presented as mean ± SD. Statistical analysis was performed using one‐way ANOVA followed by Tukey's post hoc test; different superscript letters indicate significant differences between groups. (C,D) Antioxidant activity profiles across the 40 SEC fractions derived from lemon (C) and ginger (D) EV preparations isolated by tangential flow filtration (TFF), 10% polyethylene glycol precipitation (PEG 10%), or UC, as measured by ABTS and ORAC assays. Data are presented as mean ± SD.

Analysis of antioxidant activity across fractions showed that, in general, higher activity tended to localize in contaminant‐rich fractions rather than in PDNV‐enriched fractions (Figure 7C,D). This pattern was observed for both lemon and ginger samples and across both ORAC and ABTS assays, supporting the presence of co‐isolated soluble antioxidants and proteins in non‐vesicular fractions. Notably, antioxidant activity in these fractions often exceeded that of purified nanovesicle fractions.

It is important to note that protein concentrations in several fractions were extremely low or near the detection limit. Consequently, normalization of antioxidant activity to protein content in these fractions led to artificially inflated values. Fractions with protein concentrations below 0.01 mg/mL—particularly early (F1–F4) and late (F30–F40) fractions—should therefore be interpreted with caution when applying protein‐based normalization.

#### 3.3.8 Standardized Assays Enable Consistent Interlaboratory Quantification

Interlaboratory validation of the ABTS and ORAC assays was performed using identical EV preparations derived from six plant sources (strawberry, carrot, lemon, blueberry, ginger and melon), processed in parallel in two independent laboratories following harmonized protocols (Figure 8). As summarized in Figure 8, the dataset showed generally favourable interlaboratory performance for total‐protein‐normalized TEAC values. Using the predefined pragmatic benchmark, 5 of 6 fruit‐level pairs met CV < 20% for ORAC‐PDNVs, 5 of 6 for ABTS‐PDNVs and 5 of 6 for ABTS‐PDNVs‐SDS. Correlation analyses were also supportive: ORAC‐PDNVs showed Pearson *r* = 0.98 and Spearman rho = 0.89, ABTS‐PDNVs showed Pearson *r* = 0.95 and Spearman rho = 0.89, and ABTS‐PDNVs‐SDS showed Pearson *r* = 0.90 and Spearman rho = 0.89. Figure S8 was concordant with this interpretation: scatterplots showed most fruit‐level means clustering close to the line of identity, particularly for ORAC‐PDNVs and ABTS‐PDNVs, whereas Bland–Altman plots showed only small average log‐scale biases and no obvious magnitude‐dependent drift, together supporting good cross‐laboratory transferability.

**FIGURE 8:**
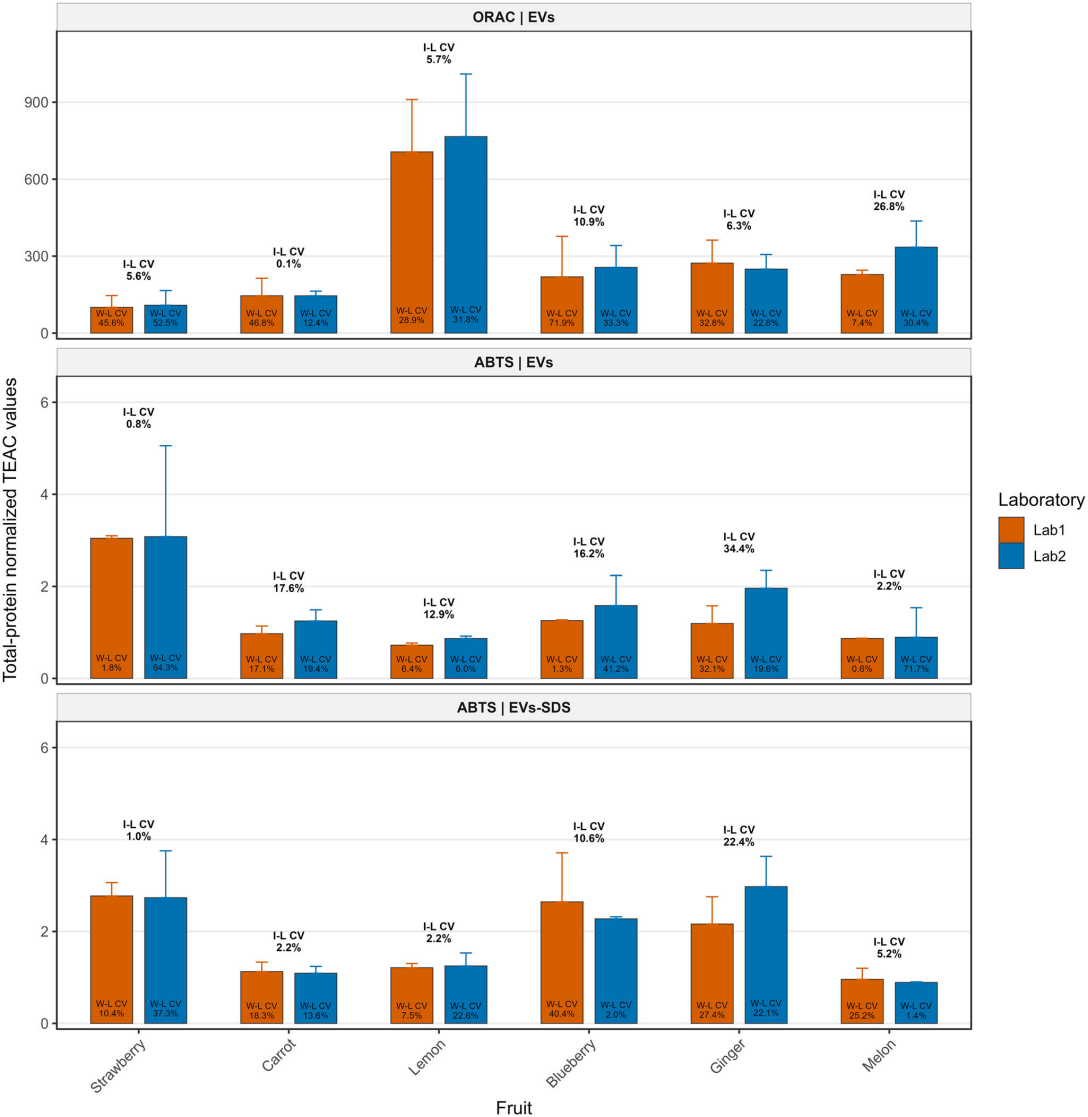
Interlaboratory validation of total‐protein‐normalized antioxidant activity measurements in PDNVs. Total‐protein‐normalized TEAC values measured in two independent laboratories for ORAC‐EVs, ABTS‐EVs and ABTS‐EVs‐SDS across six fruit‐derived preparations. Bars represent laboratory means, and upper error bars represent +SD. In‐bar labels indicate within‐laboratory coefficients of variation (W‐L CV), and top labels indicate inter‐laboratory coefficients of variation (I‐L CV) calculated from the two laboratory means for each fruit. For comparability, the ABTS‐EVs and ABTS‐EVs‐SDS panels are shown on the same y‐axis scale. Tomato and onion were excluded because total protein concentrations below 0.01 mg/mL can generate artificially magnified normalized values that are not reliable for quantitative interpretation.

Within‐laboratory repeatability was less uniform and remained assay‐, fraction‐ and matrix‐dependent. For ORAC‐PDNVs, 4 of 6 fruits met the intra‐assay CV < 15% criterion in Lab 1 and 2 of 6 in Lab 2. For ABTS‐PDNVs, 4 of 6 fruits met this threshold in Lab 1 and 1 of 6 in Lab 2, whereas for ABTS‐PDNVs‐SDS the pass counts were 2 of 6 in both laboratories. In the exploratory ABTS model, PDNVs‐SDS was not associated with a statistically consistent global shift relative to EVs after adjustment for fruit and laboratory (estimate = 0.087, *p* = 0.169), supporting the view that any SDS‐related effect is matrix‐dependent rather than uniform across fruits. Complete fruit‐level descriptive and agreement metrics are provided in Table S1. These findings demonstrate that the standardized microplate‐based ABTS and ORAC assays yield reproducible and transferable measurements of PDNV antioxidant activity, particularly when applied to highly purified nanovesicle preparations.

### 3.4 Antioxidant Activity of Plasma‐Derived EVs in Response to Dietary Intervention

The optimized assays were applied to EVs isolated from human plasma following hydroxytyrosol supplementation. (Figure 9). The plasma samples were obtained from a previously published dietary intervention study conducted by our group (Del Saz‐Lara et al. 2025), in which 11 healthy volunteers consumed hydroxytyrosol capsules daily for one week. Blood samples were collected at five time points: baseline (0 min), 30, 60, 120 min, and after one week of supplementation.

**FIGURE 9:**
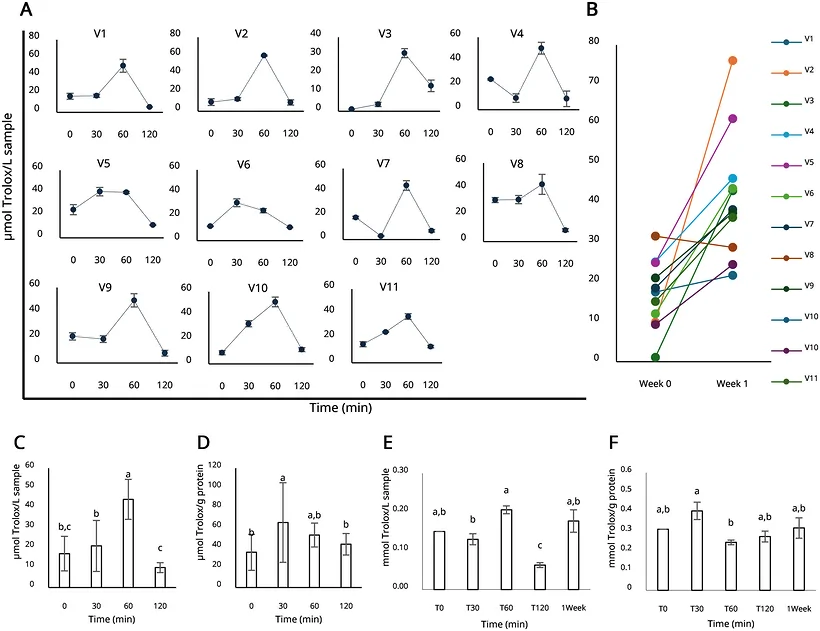
Plasma EV antioxidant activity following hydroxytyrosol supplementation in human volunteers. (A) ORAC antioxidant activity of EVs from individual volunteers at baseline and after 1 week of hydroxytyrosol capsule intake. (B) Paired baseline versus 1‐week ORAC responses for individual volunteers. (C,D) Mean plasma EV antioxidant activity measured by ORAC, shown as raw values (C) and normalized to EV protein content (D). Different letters indicate significant differences among time points by one‐way ANOVA followed by Tukey's post hoc test (*p* ≤ 0.05; *n* = 11 biological replicates per time point). (E,F) ABTS antioxidant activity of pooled plasma EV samples, shown as raw values (E) and normalized to EV protein content (F). Because ABTS was measured in pooled samples, panels E and F are descriptive and no biological inferential statistics are shown.

The ORAC assay was performed individually on EVs from each volunteer at every time point. Due to limited sample volume, the ABTS assay was conducted using pooled plasma EVs, prepared by combining 20 µL from each individual sample at each time point to generate a composite (‘macro’) sample.

#### 3.4.1 Time‐Dependent Changes in Antioxidant Activity

ORAC analysis, performed on individual plasma EV samples, showed that antioxidant activity peaked at 60 min post‐consumption (Figure 9A,C), in parallel with an increase in total EV protein content, used here as a proxy for EV abundance. In addition, most volunteers showed higher ORAC antioxidant activity after 1 week than at baseline (Figure 9B), supporting a sustained response to hydroxytyrosol supplementation.

In the pooled ABTS analysis, the highest raw antioxidant activity was also observed at 60 min (Figure 9E). When antioxidant activity was normalized to EV protein content, the highest specific activity was observed at 30 min in both the ORAC and pooled ABTS readouts (Figure 9D,F). Taken together, these data suggest that the increase in total antioxidant activity at 60 min was driven largely by EV abundance, whereas antioxidant activity per unit of EV protein was greatest at 30 min.

#### 3.4.2 Protein Content as a Normalization Metric for Extracellular Vesicles Antioxidant Activity

At baseline, a strong correlation was observed between ORAC antioxidant activity and protein content in isolated extracellular vesicles (*r* ^2^ = 0.8496; Figure S9). Although this correlation weakened across the different time points of the intervention, it did not disappear entirely. The observed reduction in the correlation coefficient may reflect genuine changes in antioxidant activity induced by the intake of a potent antioxidant such as hydroxytyrosol. When all data points from the intervention study were analyzed collectively, the overall correlation remained robust (*r* ^2^ = 0.6318; Figure S9), supporting a proportional relationship between exosome concentration (as approximated by protein content) and antioxidant capacity. This further validates protein content as a reliable proxy for EV abundance when evaluating antioxidant activity.

To address the possibility that protein content may vary across vesicles from different biological sources, we additionally performed correlation analyses using NTA particle concentration as an alternative normalization metric. In contrast to the strong correlations observed with protein content, only a weak relationship was found between ORAC antioxidant activity and particle concentration (*r* ^2^ = 0.1061; Figure S9 right panel), reinforcing the suitability of protein‐based normalization for cross‐sample comparisons within this human intervention study.

### 3.5 Antioxidant Activity of BEVs

Finally, we evaluated the applicability of the method to BEVs derived from *Pseudomonas aeruginosa*, a Gram‐negative opportunistic pathogen known to release BVs carrying virulence factors involved in host–pathogen interactions (Qin et al. 2022). BMVs were isolated by UC + SEC (Figure 10A–F).

**FIGURE 10:**
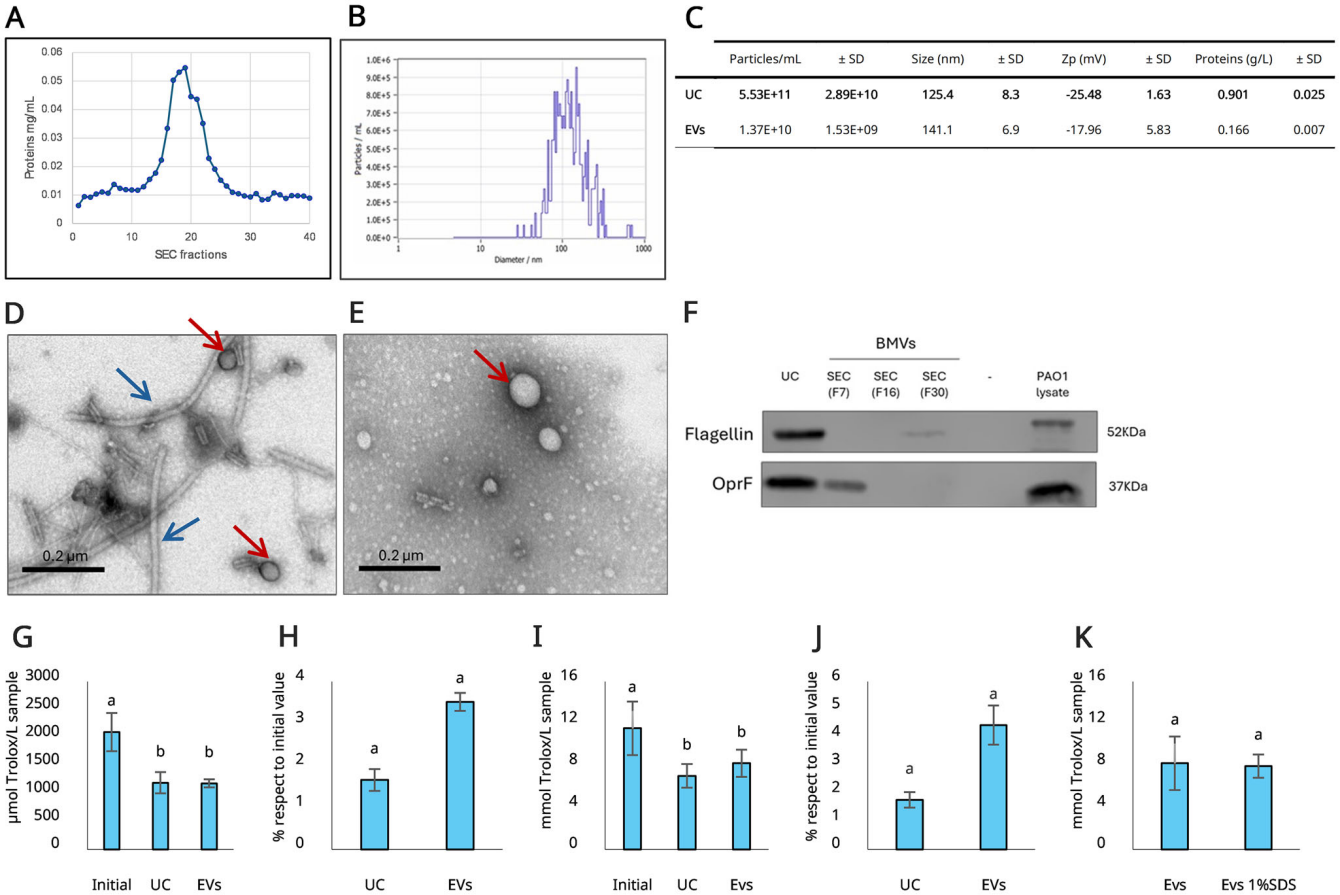
Antioxidant activity of *Pseudomonas aeruginosa* membrane vesicles (BMVs) isolated by UC or UC + SEC. (A–C), NTA size distribution, protein elution profile and mean particle characterization. (D, E) TEM morphology of BMVs isolated by UC (D) or UC + SEC (E). (F) Western blot analysis of BMVs protein marker OprF. (G, H) ORAC‐derived antioxidant activity across isolation fractions (G) and percentage retained relative to starting material (H). (I, K), ABTS‐derived antioxidant activity across fractions (I), percentage retained (J) and effect of 1% SDS disruption compared with untreated BMVs (K). ^a,b^ Different letters indicate significant differences among the samples through Tukey post‐hoc test (*p* ≤ 0.05); *n* ≥ 3.

As observed for plant‐derived EVs, purification reduced measured antioxidant activity compared to crude extracts, although BMVs retained relatively higher activity after purification (Figure 10G). However, in contrast to plant EVs, this reduction was less pronounced, and purified BMVs retained comparatively higher antioxidant activity (Figure 10H). The same trend was observed for ABTS analyses (Figure 10I, J). Consistently, the TEAC/BCA ratio was higher in BMVs than in either plant‐ or plasma‐derived EVs, assessed by either ORAC (883.33 ± 53.15 µmol/g protein) or ABTS (6.15 ± 0.97 mmol Trolox/g protein).

Unexpectedly, disruption of BMVs with 1% SDS did not further increase antioxidant activity. Instead, BMVs retained their original antioxidant capacity following lysis (Figure 10K), suggesting that encapsulated components contribute minimally to total antioxidant potential. Pyocyanin, a redox‐active pigment produced by *P. aeruginosa*, has been reported to exhibit antioxidant activity (Shouman et al. 2023). To exclude its potential contribution, we assessed its presence in purified BMVs using a previously described absorbance‐based method (Bernabe et al. 2022). Pyocyanin was not detected in purified BMV samples (data not shown), indicating that it does not account for the observed antioxidant activity. Together, these results indicate that the antioxidant properties of *P. aeruginosa* BMVs are largely attributable to membrane‐associated components, lack of antioxidant‐related cargo or both.

These findings validate the versatility and robustness of our optimized ABTS and ORAC protocols, together with the EV‐disruption assays, across diverse EV sources—from plant and plasma to bacterial BEVs—providing a reliable framework for quantifying both surface‐associated and total antioxidant activity in EVs.

#### 3.5.1 Protein Normalization for Cross‐Kingdom EV Comparisons

To assess whether protein‐based normalization can be extended to comparisons involving bacterial vesicles, we performed correlation analyses combining data from different EVs and nanovesicle sources. When PDNVs and mammalian EVs were analyzed together, a moderate correlation was observed between ORAC antioxidant activity and protein content (*r* ^2^ = 0.5829; Figure S10A), supporting the use of protein normalization for comparisons within these two kingdoms. However, when BMVs were included in the analysis, the correlation was substantially reduced (*r* ^2^ = 0.0117; Figure S10C), indicating that protein content does not serve as a reliable proxy for EV abundance across phylogenetically distant sources.

We additionally performed correlation analyses using NTA particle concentration as an alternative normalization metric. When plant and mammalian EVs were combined, a weaker relationship was found between ORAC activity and particle concentration (*r* ^2^ = 0.2949) compared to that observed with protein content. Upon inclusion of BMVs, the correlation was further diminished (*r* ^2^ = 0.027). These findings collectively suggest that while protein normalization is suitable for comparing EVs from closely related sources (e.g., plant and mammalian), it should be applied with caution—or avoided altogether—when making cross‐kingdom comparisons involving bacterial vesicles, as inherent differences in protein composition and vesicle architecture may confound the results.

### 3.6 PDNVs Exhibit Cell‐Type Dependent Redox and Viability Effects

To assess whether the antioxidant activity measured in purified PDNVs translated into biologically relevant effects, we evaluated intracellular ROS levels in EA.hy926 endothelial cells after a short exposure period (4 h) (Figure 11A). ROS levels and cell viability was also evaluated after longer exposure (24 h) in THP‐1 monocytes, EA.hy926 endothelial cells, and Caco‐2 intestinal epithelial cells using assay‐specific concentration ranges (Figure S11 and Figure 11B).

**FIGURE 11:**
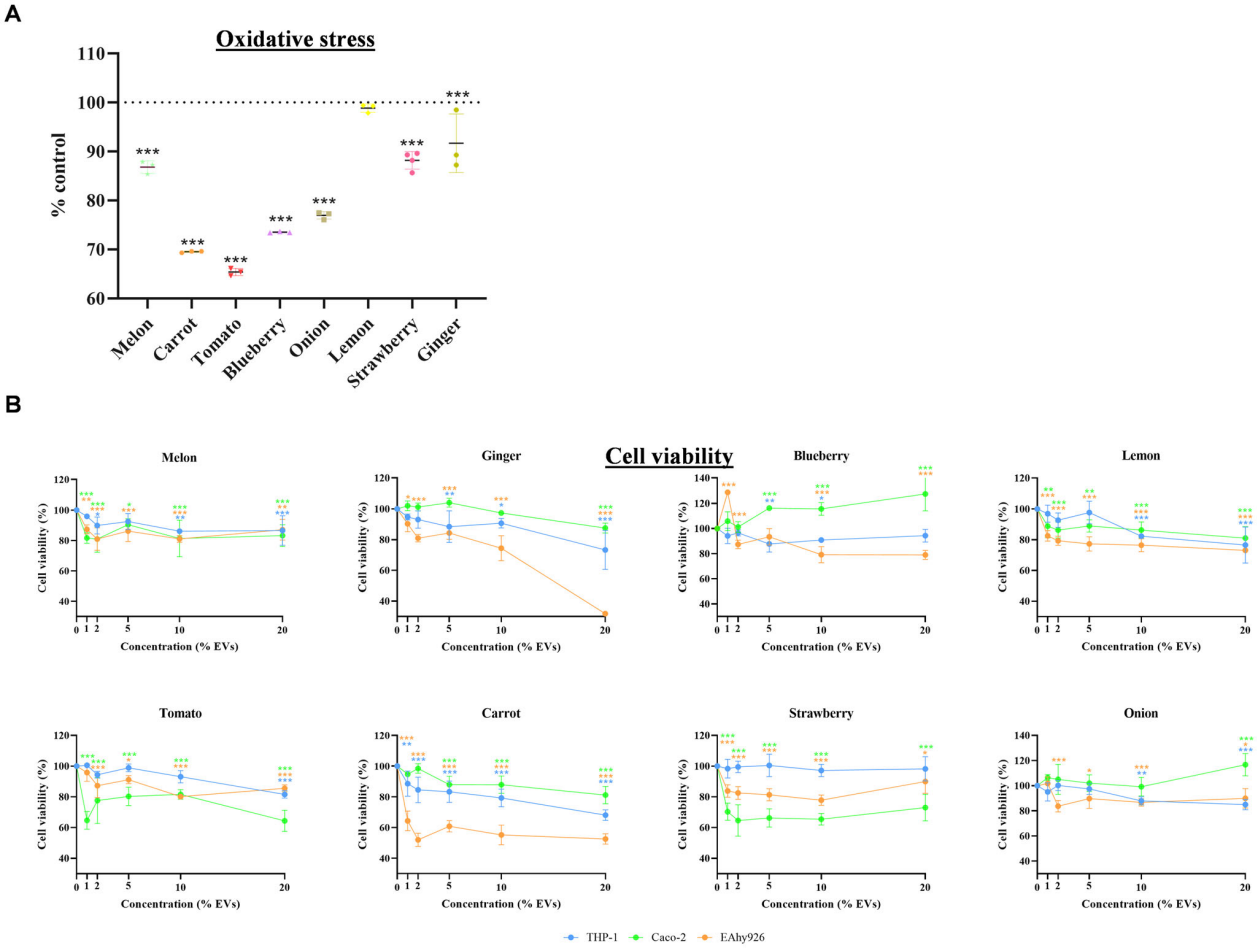
Cell‐type‐dependent effects of PDNVs on intracellular ROS levels and cell viability. THP‐1 monocytes, EA.hy926 endothelial cells, and Caco‐2 intestinal epithelial cells were treated with PDNVs from different botanical sources. (A) Intracellular ROS levels in EA.hy926 endothelial cells after treatment (4 h) with PDNVs at the concentrations of 10% or lower, expressed as mean fluorescence intensity (MFI) relative to untreated control cells (set to 100%). (B) Cell viability after treatment (24 h) with PDNVs at the concentrations indicated in panel B, expressed as percentage of untreated control cells. Data are presented as mean ± SD from at least three independent experiments performed in technical triplicate. Statistical significance was assessed by one‐way or two‐way ANOVA, as appropriate, followed by Dunnett's post hoc test for multiple comparisons versus control. Significance is indicated by *p < 0.05, **p < 0.01 and ***p < 0.001.

At 4 h, all PDNEV preparations reduced intracellular ROS levels in EA.hy926 endothelial cells to different extents (Figure 11A). Preparations displaying higher antioxidant activity in the ORAC and ABTS assays generally produced a greater reduction in cellular ROS. Correlation analysis between chemical antioxidant activity and intracellular ROS demonstrated a moderate inverse relationship for both ORAC (*R* ^2^ = 0.301) and ABTS (*R* ^2^ = 0.3797) (Figure S12), indicating that EV preparations with higher in vitro antioxidant capacity generally exerted stronger antioxidant effects in cells.

However, at 24 h, ROS measurements revealed clear cell‐type‐ and source‐dependent responses (Figure S11). In THP‐1 monocytes, most EV preparations produced modest changes around control values, although ginger‐derived EVs tended to reduce ROS at some concentrations. EA.hy926 endothelial cells showed the most pronounced and heterogeneous responses, with several EV preparations markedly increasing ROS levels, whereas ginger‐derived EVs reduced ROS below baseline at intermediate concentration. In Caco‐2 intestinal epithelial cells, ROS responses were generally more moderate and variable, with both increases and slight reductions depending on EV source and concentration.

Cell viability was also modulated in a cell‐ and source‐dependent manner (Figure 11B). THP‐1 and Caco‐2 cells were generally well tolerated across most conditions, whereas EA.hy926 cells showed greater sensitivity to selected PDNV preparations, particularly at higher concentrations. Overall, the strongest changes in ROS were not uniformly accompanied by parallel changes in viability across all cell types and nanovesicle sources.

These findings indicate that ORAC and ABTS measurements should be interpreted as screening indicators associated with early ROS modulation under the conditions evaluated, rather than as direct surrogates of cellular antioxidant efficacy, which additionally depends on biological processes such as vesicle uptake, intracellular trafficking, metabolism and adaptive redox responses.

## 4 Discussion

EVs are increasingly recognized as key mediators of redox homeostasis, capable of modulating oxidative stress through the transfer of bioactive molecules, including antioxidant enzymes, lipids and regulatory RNAs. However, despite growing interest in their antioxidant properties, quantitative assessment of EV‐associated antioxidant activity remains poorly standardized. In this study, we establish and validate a methodological framework that addresses critical limitations in the field, including low EV input, co‐isolated contaminants and assay‐specific artifacts.

### 4.1 Methodological Optimization and Sensitivity

While microplate‐based ABTS (Kut et al. 2022; Yu et al. 2022) and DPPH (Herald et al. 2012; Musa et al. 2013) protocols exist, to our knowledge, ours is the first systematic effort to tailor these methods for the unique challenges of EV analysis, particularly their low abundance and potent antioxidant potential. Among the single electron transfer (SET)‐based assays (ABTS and DPPH), only the modified ABTS protocol proved suitable for EVs. The miniaturized assay with 25 µL sample input maintained sensitivity while accommodating the low yields of SEC‐purified EVs. In contrast, the DPPH assay showed non‐linear responses and strong optical interference, making it unreliable for EVs despite its frequent use in single‐step EV preparations (Li et al. 2023; Ma et al. 2024).

Our comparative analysis of ABTS, DPPH and ORAC assays reveals that not all antioxidant assays are suitable for EV analysis. While DPPH has been widely used in the literature—including for bacterial vesicles—we found it to be insufficiently sensitive and prone to interference in low‐input EV samples (Wang et al. 2025). In contrast, ABTS and ORAC provide complementary information: ABTS captures total electron‐transfer capacity, whereas ORAC reflects hydrogen atom transfer reactions and is more representative of physiological radical scavenging.

### 4.2 Mechanistic Insights From ORAC Assays

Unlike Trolox or free phenolics, which displayed rapid depletion profiles, EVs showed a progressive decline in fluorescence, suggesting a continuous exchange of fluorescein and radicals across vesicle membranes (Adamo et al. 2025). This observation implies that EVs not only act as carriers of antioxidant molecules but may also modulate radical reactions through their structural properties. Furthermore, because ORAC is based on peroxyl radicals—a physiologically relevant oxidant—our results suggest it may provide a more biologically representative assessment of EV antioxidant capacity than ABTS, which relies on a synthetic radical (Munteanu and Apetrei 2021). However, its differential mechanism for neutralizing free radicals (i.e., hydrogen atom transfer, HAT) need to be considered.

A key optimization was the implementation of a sample‐specific *self‐blank* to correct for an initial quenching of fluorescence caused by EV addition, a phenomenon overlooked in previous EV‐ORAC studies (Lee et al. 2023; Yu et al. 2022), that would lead to significant underestimation of activity. Moreover, the greater sensitivity of ORAC also enabled detection of low‐activity EVs that were undetectable by ABTS, supporting its preferential use when sample availability or activity is limiting. Briefly, we recommend ABTS for studies focused on total biochemical payload (requiring membrane disruption), whereas ORAC is preferred for assessing the biological radical‐scavenging potential of intact vesicles.

A key methodological advance in this study is the introduction of a self‐blank correction in ORAC assays, which accounts for EV‐induced fluorescence quenching. This previously unrecognized artifact may lead to systematic underestimation of antioxidant activity and could explain variability across studies. Together, these results highlight that EV antioxidant activity is not an intrinsic single parameter, but rather an assay‐dependent property that requires careful methodological calibration.

### 4.3 Methodological Limitations in the Field and Need for Standardization

A major challenge in interpreting EV antioxidant activity arises from the heterogeneity of isolation methods and the frequent lack of rigorous purification. Previous studies—particularly in plant‐derived EVs—often report high antioxidant activity using crude extracts or minimally purified samples. Our results demonstrate that such approaches substantially overestimate EV‐associated antioxidant capacity, as most of the measured activity originates from soluble, non‐vesicular components. Importantly, this conclusion is further supported by our analysis of extracellular vesicles isolated from AWF, which represents a minimally disruptive approach to obtain plant EVs and is considered closer to native extracellular vesicle populations. In contrast to vesicles derived from mechanically processed tissues, AWF‐derived EVs exhibited very low antioxidant activity following SEC purification, despite measurable activity in earlier fractions. This observation reinforces that the high antioxidant capacity often reported in plant‐derived EV preparations is largely attributable to co‐isolated soluble components released during tissue disruption. The inclusion of AWF‐derived EVs therefore provides an important biological reference, supporting that bona fide plant EVs possess limited intrinsic antioxidant activity under basal conditions. These findings are consistent with MISEV recommendations emphasizing rigorous purification and careful interpretation of EV‐associated functions, particularly in complex biological matrices such as plant tissues.

Importantly, our work underscores a paramount, often overlooked, confounding factor in EV antioxidant studies: purification rigor. We observed a drastic reduction (>90%) in antioxidant activity between the initial extract, the UC pellet and the final SEC‐purified EVs. These findings strongly suggest that antioxidant measurements in UC‐only samples are substantially influenced by co‐isolated soluble antioxidants and other non‐vesicular components. However, we cannot exclude that purification also reduces physiologically associated EV components, including biomolecular corona constituents, or selectively depletes EV subpopulations with higher antioxidant activity. This is a crucial distinction, as many previous studies reporting high EV antioxidant activity relied solely on UC or PEG‐based precipitation without subsequent purification (Gao et al. 2025; Jang et al. 2023; Kilasoniya et al. 2023; Ma et al. 2024), likely overestimating true EV‐derived activity. This is consistent with recent calls to include SEC as a critical purification step to ensure structural and biochemical integrity of EVs (Kim et al. 2023) or the use of two different consecutive methods (i.e., MISEV guidelines 2023 (Welsh et al. 2024)) to obtain highly purified EVs.

An additional consideration is that highly stringent purification procedures, including SEC, may partially remove the biomolecular corona associated with EVs. Increasing evidence indicates that the EV corona comprises adsorbed proteins, metabolites and other soluble biomolecules that may contribute to EV stability, biodistribution and biological activity. Consequently, although SEC effectively minimizes contamination by free soluble antioxidants, it may also reduce physiologically associated corona components that contribute to the overall antioxidant properties of native EVs. Therefore, the antioxidant activity measured in highly purified EV preparations should be interpreted as the intrinsic activity of the purified vesicles rather than the complete antioxidant potential of EVs within their native biological milieu. Dedicated studies specifically addressing the contribution of the EV corona will be required to fully resolve this issue.

Beyond methodological optimization, our data provide insight into the mechanistic basis of EV antioxidant function. EVs are known to carry enzymatic antioxidants such as superoxide dismutase, catalase and peroxiredoxins, as well as non‐enzymatic molecules including lipids and small metabolites. However, the relative contribution of these components has remained unclear.

Using detergent‐based disruption, we demonstrate that EV antioxidant activity could be derived from both the surface‐associated activity, accessible in intact vesicles and likely mediated by membrane lipids or surface‐bound proteins, as well as, from encapsulated cargo activity, revealed upon vesicle disruption.

The limited increase in antioxidant activity upon disruption in most native EVs suggests that surface components dominate the measurable antioxidant capacity under physiological conditions. In contrast, cargo‐loaded EVs show enhanced activity after lysis, indicating that encapsulated antioxidants may contribute primarily upon vesicle uptake or degradation in recipient cells. This model aligns with current understanding of EV‐mediated redox signalling, where vesicles can both directly scavenge reactive species and deliver functional antioxidant machinery to target cells.

### 4.4 EV Integrity and Contribution of Internal Cargo

While the physiological impact of EVs in in vivo models likely involves the combined activity of both surface and internal components, such as antioxidant enzymes, the ability to distinguish these two pools is a critical requirement for the biochemical characterization and standardization of EV‐based products. Our disruption protocol using 1% SDS was optimized to ensure compatibility with the ABTS assay while providing sufficient lysis to unmask encapsulated cargo, as demonstrated by the significant increase in activity in phenolic‐loaded vesicles. Intact EVs display primarily surface‐driven antioxidant activity, whereas membrane lysis exposes encapsulated bioactives that substantially enhance antioxidant capacity, particularly in phenolic‐enriched EVs. These findings agree with previous work showing EVs as carriers of phenolics, vitamins and isothiocyanates (Deng et al., 2017; Zhuang et al. 2015; Zhu et al. 2022). Notably, detergent‐based disruption was compatible with ABTS but not with ORAC, where interference with fluorescein and AAPH precluded reliable measurements. We recognize that for certain rigid vesicle types, further optimization of lysis conditions or the physical separation of released cargo from membrane debris could provide additional mechanistic depth; however, the current protocol successfully achieves the primary goal of quantifying the total potential antioxidant activity in a high‐throughput format.

In addition to plant‐ and plasma‐derived vesicles, we extended our analysis to BMVs secreted by *Pseudomonas aeruginosa*. To our knowledge, no prior studies have evaluated the antioxidant activity of bacterial EVs. Our findings revealed that BMVs retained comparatively higher antioxidant activity than plant or plasma EVs when normalized to protein content, but this activity was unaffected by detergent‐mediated disruption, indicating a primarily membrane‐associated origin. This contrasts with plant EVs, where encapsulated cargo can substantially contribute to total potential antioxidant capacity. These results highlight both the versatility of our optimized protocols and the distinct mechanistic basis of antioxidant activity across evolutionary lineages of EVs.

Because lysis efficiency was not directly verified under the ABTS‐compatible conditions used for BMVs, incomplete membrane disruption or partial inactivation of encapsulated antioxidant cargo cannot be completely excluded. Nevertheless, the absence of increased antioxidant activity following detergent treatment suggests that any contribution from encapsulated components is limited under the conditions employed.

### 4.5 Biological Variability and Normalization

Antioxidant activity varied markedly across EVs from different botanical sources, with ginger, broccoli and strawberry showing the highest TEAC values, which likely reflects differences in EV composition, including protein, lipid and metabolite cargo. These results are in line with their known enrichment in bioactive phytochemicals (Deng et al. 2017; Zhuang et al. 2015, Zhang et al. 2016; Zhu et al. 2022). However, direct comparison with previous reports proved difficult due to heterogeneous reporting units (e.g., % inhibition, Trolox/mL, alternative standards) (Gao et al. 2025; Ma et al. 2024; Jang et al. 2023; Kilasoniya et al. 2023), which likely reflects differences in EV composition, including protein, lipid and metabolite cargo. Our findings support protein‐based normalization (mmol Trolox/g protein) as a proxy for EV abundance in purified samples. This metric correlated strongly with TEAC values (*r* ^2^ = 0.8497 for ABTS) and enabled meaningful comparisons across sources and methods. Regarding normalization, most fruit‐level comparisons fell below the predefined pragmatic interlaboratory benchmark, and preservation of fruit ranking was strong across all three analytical blocks. This combination of favourable cross‐laboratory agreement and stable rank order supports the workflow as a transferable comparative platform for total‐protein‐normalized TEAC assessment, particularly for ORAC‐EVs and, to a slightly more heterogeneous extent, for ABTS‐based measurements.

At the same time, the more variable within‐laboratory repeatability indicates that analytical precision is not yet uniformly optimized across assays, fractions and matrices. The exclusion of tomato and onion is also important, because it highlights a practical limitation of protein normalization when the denominator becomes too small. In line with current recommendations emphasizing rigor and transparent reporting in EV studies (Welsh et al. 2024), the present data support robust interlaboratory applicability for the analyzable matrices while also indicating that tighter control of low‐protein samples and within‐laboratory execution would be needed if stricter analytical precision is sought. To our knowledge, this is the first standardized framework for reporting antioxidant activity of purified EVs, which could facilitate reproducibility and meta‐analysis across studies. Importantly, the proposed normalization approach addresses a key limitation in the current literature, namely the lack of consistent normalization strategies for expressing EV antioxidant activity.

It should also be noted that the present interlaboratory validation was performed exclusively using representative plant‐derived nanovesicle preparations. Although these results demonstrate good transferability of the analytical workflow within this sample class, additional validation using mammalian and bacterial EVs will be required to establish the general applicability of the protocol across diverse vesicle types. Furthermore, while cross‐laboratory agreement was generally good, within‐laboratory repeatability remained matrix‐ and assay‐dependent, indicating that additional optimization may further improve analytical precision.

### 4.6 Application to Human Plasma EVs

The application of our methodology to a human dietary intervention demonstrates that EV antioxidant capacity is dynamically regulated in vivo.

The observed increase following hydroxytyrosol intake supports the concept that EVs can act as carriers of diet‐derived bioactive molecules (Arola‐Arnal et al. 2020; Chapado et al. 2021; Iglesias‐Aguirre et al. 2022) or as mediators of systemic redox adaptation. This is consistent with emerging evidence that EV composition and function are modulated by physiological stimuli, including exercise, diet and disease states (Lopez de Las Hazas et al. 2021; Mantilla‐Escalante et al. 2019; Mantilla‐Escalante et al. 2021).

Both ABTS and ORAC revealed dynamic increases in the antioxidant activity of EVs following hydroxytyrosol ingestion, peaking between 30 and 60 min. These changes reflect the pharmacokinetics of key plasma metabolites encapsulated in EVs, namely hydroxytyrosol 3‐sulfate and hydroxytyrosol 4'‐sulfate (de Las Hazas et al. 2016; Del Saz‐Lara et al. 2025). Moreover, the sustained antioxidant activity observed after one week coincides with the reduction in LDLox reported in the human study (Del Saz‐Lara et al. 2025), suggesting that plasma EV antioxidant activity may contribute to the modulation of relevant functional targets. Overall, this provides the first evidence, to our knowledge, that the antioxidant capacity of EVs can be modulated by diet and detected using standardized in vitro assays. Moreover, these results demonstrate the applicability of our methods to complex biological fluids and their sensitivity to dynamic physiological changes.

Together, these findings establish a methodological framework for reliably quantifying antioxidant activity in EVs, clarify the limitations of conventional approaches, and reveal new mechanistic insights into EV‐mediated antioxidant behaviour.

An important limitation is that lipoprotein contamination was not directly assessed in the plasma EV preparations. Because lipoproteins carry antioxidant molecules and may partially co‐isolate with extracellular vesicles, their contribution to the measured antioxidant activity cannot be completely excluded. Likewise, the absence of a placebo or time‐matched control intervention precludes complete exclusion of circadian or postprandial influences. Therefore, although the observed temporal changes are consistent with hydroxytyrosol pharmacokinetics, future placebo‐controlled studies employing additional lipoprotein characterization will be important to further validate these observations.

### 4.7 Implications for Therapeutic and Functional Applications

The ability of EVs to modulate oxidative stress has generated significant interest in their use as therapeutic agents or delivery systems. EVs offer several advantages over conventional antioxidants, including protection of labile molecules, targeted delivery and reduced off‐target effects. However, our findings indicate that accurate assessment of EV antioxidant activity requires rigorous methodological control, particularly to avoid confounding effects from co‐isolated compounds.

By providing a standardized framework for antioxidant measurement, this work contributes to improving reproducibility and comparability across studies. For that, we have generated a standardized roadmap for assessing antioxidant activity in extracellular vesicles (Figure 12). This is particularly relevant for the development of EV‐based therapeutics, where functional characterization is essential for regulatory approval and clinical translation. In overall, all the studies should indicate the EV origin and purification method, the normalization parameters, use the self‐blank correction, identify if some disruption methods were employed (to discuss if they were analyzed their cargo or surface).

**FIGURE 12:**
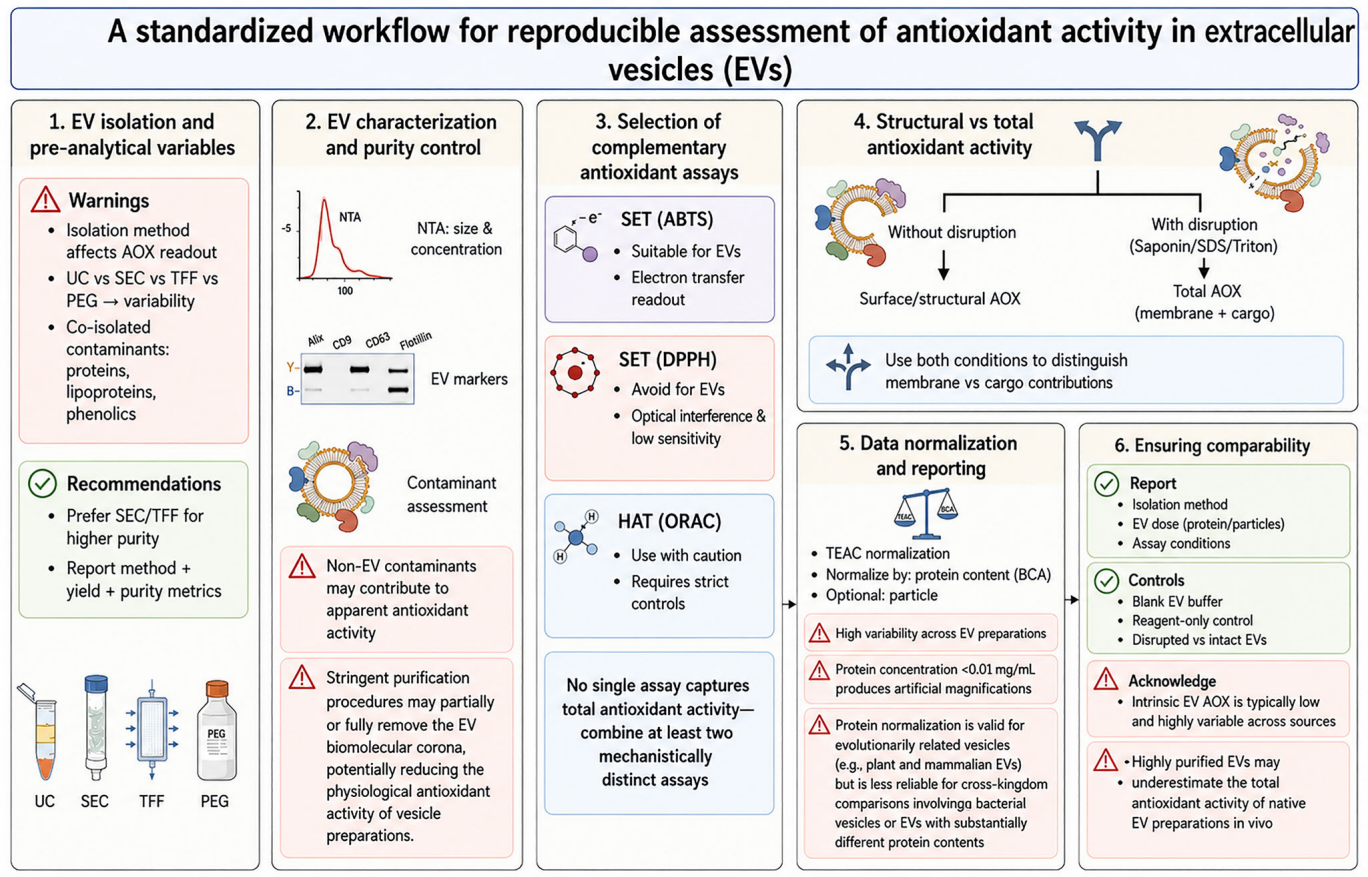
Standardized roadmap for assessing antioxidant activity in extracellular vesicles. This schematic outlines key steps and recommendations to ensure reproducible and comparable evaluation of antioxidant activity in EVs. Pre‐analytical variables, including EV isolation methods, critically influence AOX measurements due to differences in purity and co‐isolated contaminants. Rigorous EV characterization is therefore essential prior to functional assessment. Selection of complementary assays based on distinct chemical mechanisms (e.g., single electron transfer and hydrogen atom transfer) is recommended, as no single method fully captures AOX capacity. Sample preparation should distinguish between structural (membrane‐associated) and total AOX activity through controlled disruption protocols to assess the contribution of EV cargo. Given the typically low and variable intrinsic AOX activity of EVs, appropriate normalization strategies (e.g., protein content or particle number) are required. Finally, standardized reporting of experimental conditions and inclusion of adequate controls are essential to enable cross‐study comparisons and advance the field towards harmonized methodologies. Figure created with BioRender.com.

### 4.8 Limitations and Biological Considerations

While this study establishes a robust and analytically validated framework, several limitations should be considered. First, antioxidant activity was primarily assessed using chemical assays, which may not fully capture biological functionality in cellular contexts. To complement these measurements, we incorporated a cell‐based ROS assay together with cell‐viability assessment in multiple cell models. These experiments showed that purified EVs are biologically active and capable of modulating intracellular redox status, but that these effects are strongly dependent on EV source, dose and recipient cell type.

Importantly, the lack of a uniform correspondence between cell‐free antioxidant capacity, ROS modulation and viability indicates that chemical antioxidant readouts should be interpreted as comparative properties of EV preparations rather than as direct predictors of cytoprotective outcome. Thus, the cellular data strengthen the biological relevance of the proposed methodology while also underscoring that EV‐associated redox activity is context dependent.

The molecular determinants of antioxidant activity—such as specific enzymes, lipids, RNAs or metabolites—were not directly identified and warrant further investigation using omics approaches. The analytical performance of the ORAC assay was further supported by determination of the LoD and quantification (LoQ), together with spike‐in recovery experiments performed using representative PDNVs. These analyses demonstrated adequate sensitivity for highly purified PDNV preparations and negligible matrix interference under the experimental conditions employed. Nevertheless, additional analytical validation across other EV sources and more complex biological matrices would further strengthen the broader applicability of the method. We recognize additional limitations and considerations. The ABTS and ORAC assays used in this study are in vitro models that do not fully mimic physiological conditions. However, they remain widely accepted benchmarks for measuring antioxidant capacity and offer a practical starting point for screening EV functionality. The detergent‐based disruption technique helps differentiate between surface‐bound and cargo‐derived activity, although complete separation cannot be guaranteed across all EV types. Nonetheless, this method provides a useful comparative framework and highlights structure–function relationships within EVs. Protein normalization was employed to account for sample variability, though it may not fully reflect functional equivalence across different EV populations, particularly when comparing vesicles from different biological kingdoms. Likewise, although purification substantially reduced the contribution of soluble non‐vesicular antioxidants, it may also partially remove physiologically associated biomolecular corona components or selectively deplete EV subpopulations with distinct biochemical properties. Consequently, the antioxidant activity reported here should be interpreted as the intrinsic activity of highly purified EVs rather than the complete antioxidant potential of native vesicles in their physiological microenvironment. Despite these limitations, it enables reproducible quantification and comparison across studies. Additional methodological limitations should also be acknowledged. The interlaboratory validation was performed using representative PDNVs and therefore remains to be extended to mammalian and bacterial EVs. In addition, lipoprotein contamination was not directly assessed in plasma EV preparations, and therefore a contribution from co‐isolated plasma components cannot be completely excluded. Likewise, because a placebo or time‐matched control intervention was not included, circadian or postprandial effects cannot be entirely ruled out. Finally, although detergent treatment did not increase the antioxidant activity of *Pseudomonas aeruginosa* BMVs, lysis efficiency was not directly verified under the ABTS‐compatible conditions employed, and incomplete membrane disruption cannot be fully excluded. Future research combining complementary assays and in vivo validation will be necessary to further elucidate the biological significance of EV antioxidant properties.

## 5 Conclusions

We have developed and analytically validated optimized microplate‐ based ABTS and ORAC assays tailored for the measurement of antioxidant activity in highly purified EVs, applicable to plant, human plasma and bacterial sources. Our results demonstrate that (i) rigorous purification is essential to minimize overestimation of EV antioxidant activity arising from co‐isolated soluble components, although highly stringent purification may also partially remove physiologically associated EV corona components; (ii) normalization to protein content (mmol Trolox/g protein) provides a practical and widely accessible metric that correlates strongly with EV abundance in purified samples and facilitates comparisons within comparable EV populations, while cross‐kingdom comparisons should be interpreted with appropriate caution; (iii) intact EVs primarily exhibit surface‐based activity, with enhanced capacity upon cargo release; and (iv) the optimized ORAC assay, supported by LoD/LoQ determination and spike‐in recovery experiments, provides high analytical sensitivity with minimal matrix interference and is particularly well suited for detecting the low antioxidant activity of highly purified EV preparations. However, the ABTS assay is indispensable for assessing total antioxidant potential when combined with a validated disruption protocol. Importantly, we show for the first time that EV antioxidant activity can be modulated in vivo by dietary supplementation, and that BMVs from *P. aeruginosa* also display measurable antioxidant properties, representing the first report of this phenomenon in bacterial EVs.

Collectively, these methodological advances, together with analytical validation and supporting cell‐based assessment of EV‐mediated redox effects, provide a standardized and reproducible framework for quantifying EV‐associated antioxidant activity across diverse biological sources while facilitating future mechanistic and translational studies.

## Author Contributions

**Juan Antonio Nieto**: conceptualization, investigation, Writing – original draft, methodology, validation, visualization, data curation, formal analysis. **María‐Carmen López de las Hazas**: conceptualization, funding acquisition, supervision, writing – review and editing. **Victoria Martín‐santiago**: investigation, validation, formal analysis, visualization. **Laura Rubio‐silvan**: investigation, methodology, validation, formal analysis, visualization. **Carmen Mazarío‐gárgoles**: investigation, validation, formal analysis, visualization. **Ludovica Attanasio**: investigation, methodology. **Benedetta Mercuriali**: investigation, validation, visualization, formal analysis. **João Tomé‐carneiro**: writing – review and editing, methodology, supervision. **Giulia Bernabè**: investigation, methodology. **Andrea del Saz‐lara**: investigation. **Carmen Crespo**: investigation. **María Ángeles Ávila‐gálvez**: investigation. **Emma Burgos‐ramos**: investigation. **Juan Carlos Espín**: investigation. **Andrea Chini**: investigation, validation. **Alberto Dávalos**: conceptualization, investigation, funding acquisition, writing – original draft, methodology, writing – review and editing, project administration, supervision, resources.

## Conflicts of Interest

The authors declare no conflicts of interest.

## Supporting information




**Supporting Information**: jev270392‐sup‐0001‐SuppMat.pdf

## Data Availability

The data that support the findings of this study are available from the corresponding author upon reasonable request.
