# Supplementary material for: Standardized Microplate‐Based Assays for Quantifying Antioxidant Activity in Extracellular Vesicles and Nanovesicles Across Biological Kingdoms
Source: J Extracell Vesicles. 2026 Nov 3;15(11):e70392. doi: 10.1002/jev2.70392 (PMC13730445; doi:10.1002/jev2.70392)
Supplement: Supplementary file 1 [file JEV2-15-e70392-s001.pdf]

Supplementary Information

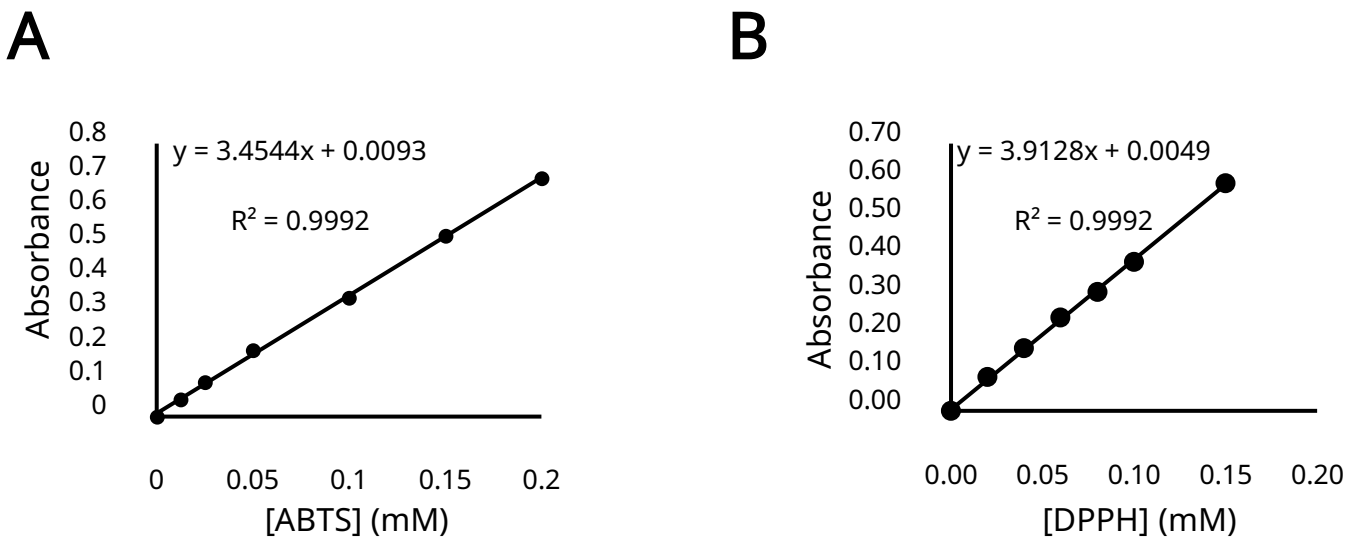

Figure S1. ABTS radical (A) and DPPH radical (B) standard curve.

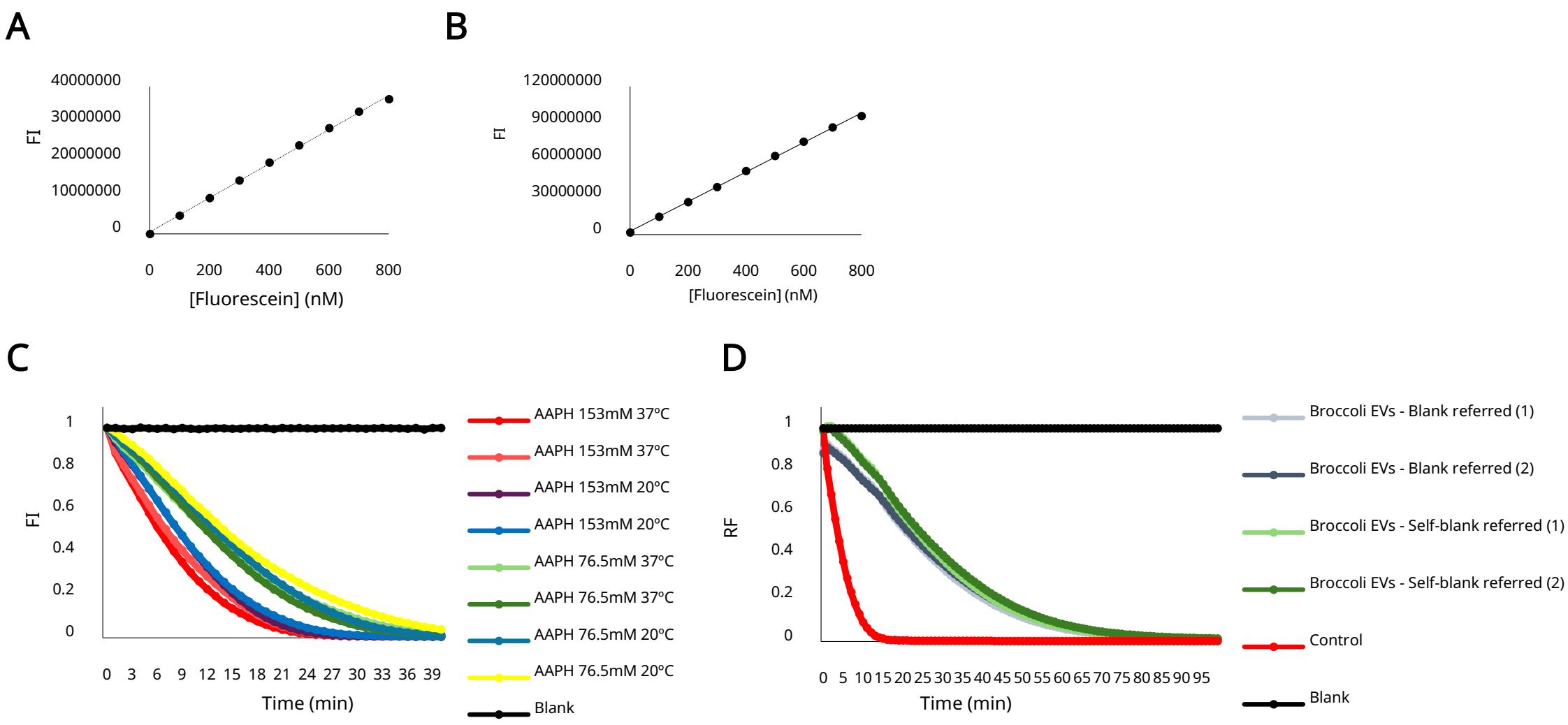

Figure S2. Optimization of the fluorescein solution and AAPH solution for the ORAC method adapted to EV samples. (A) Fluorescein standard curve [fluorescence intensity (FI) versus fluorescein concentration] at 460 nm excitation and 530 nm emission. (B) Fluorescein standard curve at 480 nm excitation and 530 nm emission. (C) Optimization of the AAPH solution by evaluating AAPH concentration and temperature conditions. (D) Effect of blank correction on relative fluorescence (RF) calculation throughout the assay.

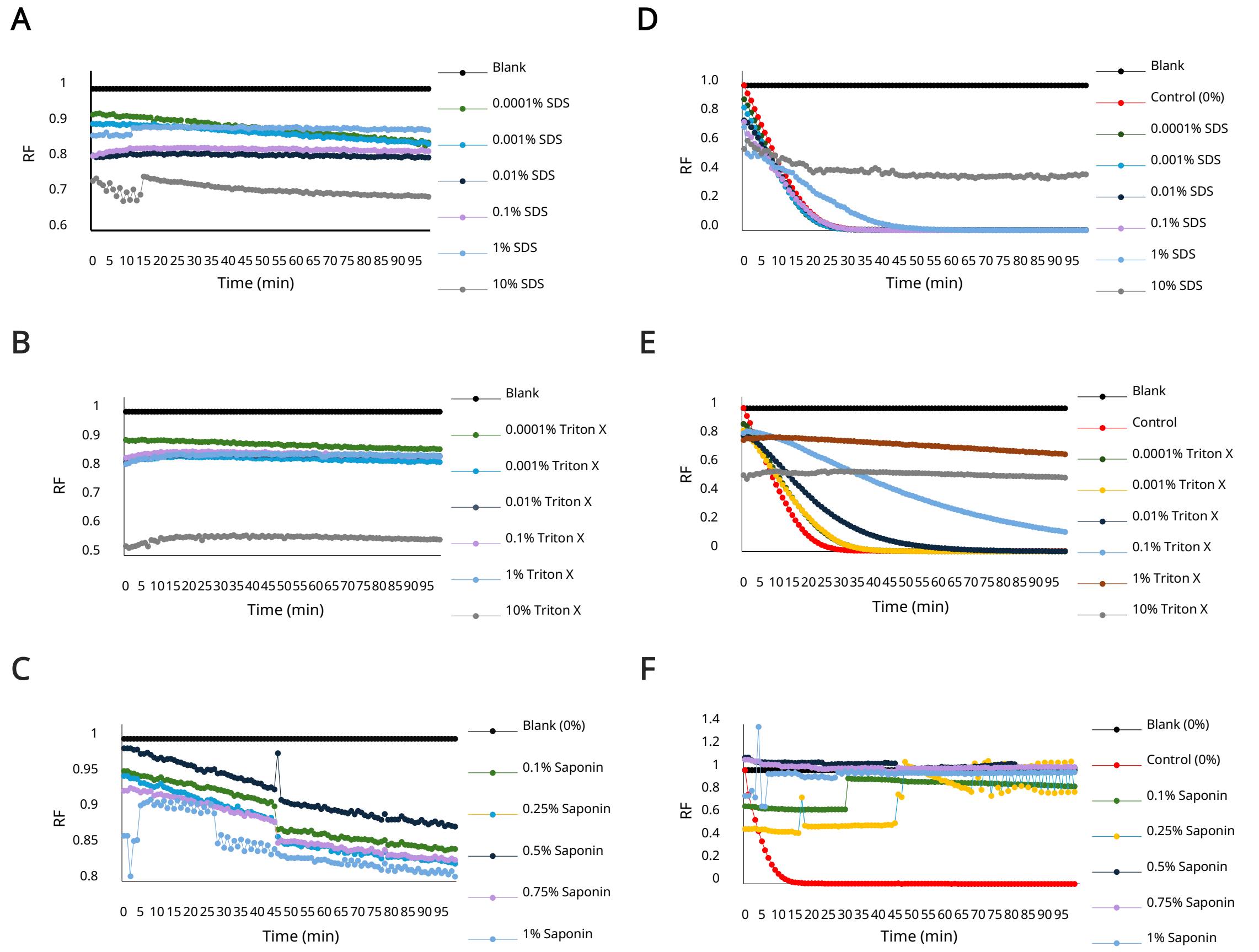

Figure S3. **Determination of potential interferences of SDS, Triton X-100 and saponin on ORAC method**. A. Influence of SDS concentration in fluorescein intensity along the time (expressed as relative fluorescence (RF)), B. Triton X-100 concentration effect, and C. saponin concentration effect. D. Influence of SDS concentration in AAPH activity against fluorescein in ORAC method (expressed as relative fluorescence (RF)), E. Triton X-100 concentration effect, and F. saponin concentration effect.

Sample	Gallic acid (µM)	Resveratrol (µM)	Quercetin (µM)	Hydroxytyrosol (µM)
EVs Control	ND	ND	ND	ND
EVs + Gallic acid	<LOQ	ND	ND	ND
EVs + Resveratrol	ND	0.15	ND	ND
EVs + Quercetin	ND	ND	0.27	ND
EVs + Hydroxytyrosol	ND	ND	ND	0.23

Figure S4. Loading of dietary polyphenols into broccoli-derived EVs. Loading was performed by passive diffusion. Polyphenols were extracted from 200 µL of EVs and analyzed by UPLC-qTOF-MS to quantify their concentrations. Gallic acid LOQ = 0.01 µM. ND, not detected; LOQ, limit of quantification.

A

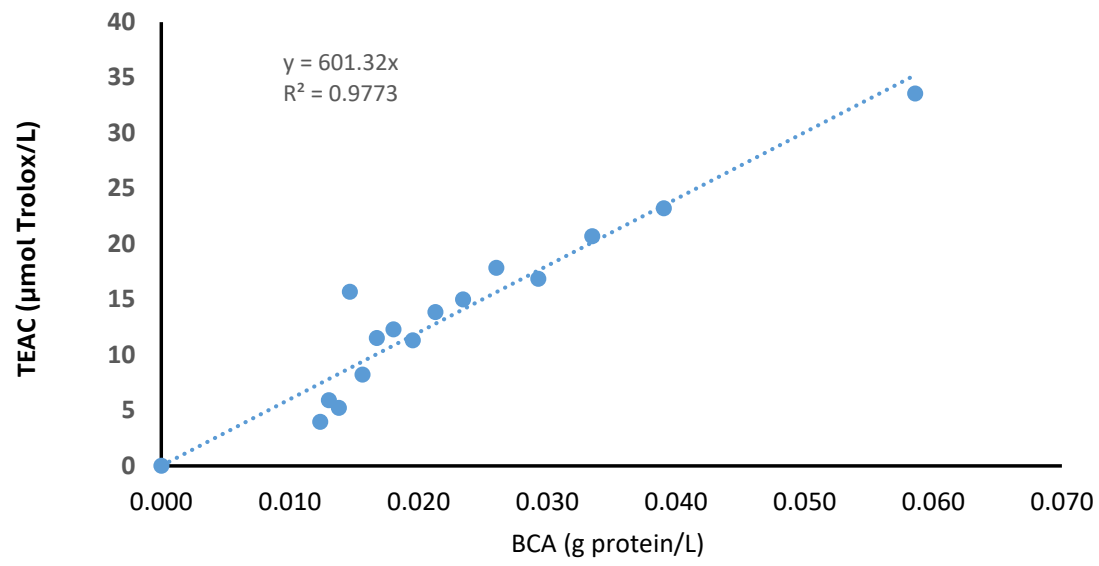

B

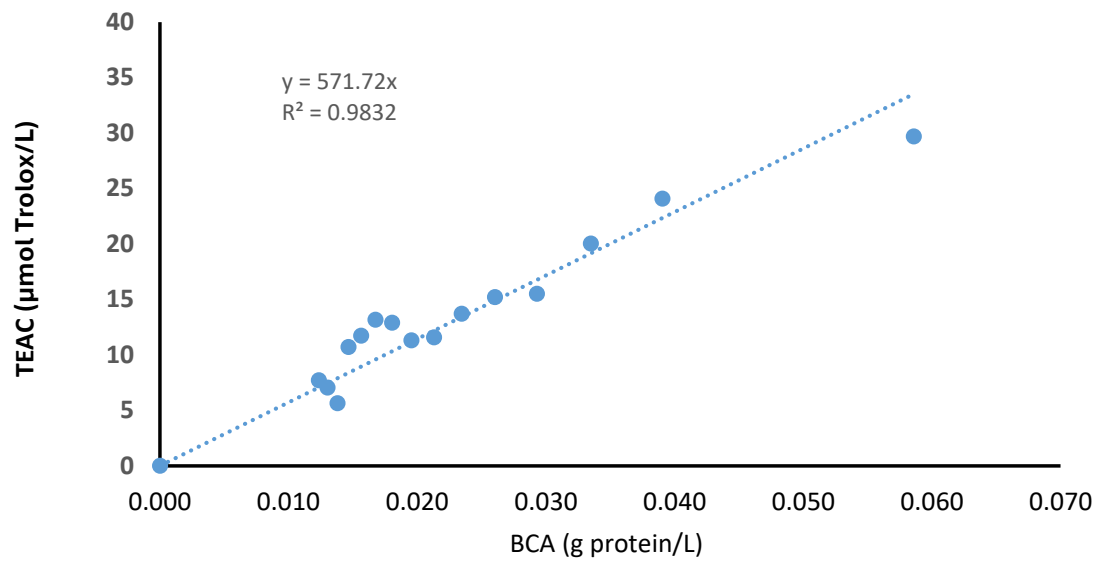

C

	**Laboratory 1**		**Laboratory 2**	
**PDNVs**	**L.O.D.**	**L.O.Q.**	**L.O.D.**	**L.O.Q.**
Lemon	0.013813928	0.041860389	0.01211669	0.036717242
Melon	0.134501684	0.40758086	0.119912997	0.363372718
Blueberry	0.004480186	0.01357632	0.002594241	0.007861337

Figure S5. **Limit of detection (L.O.D) and limit of quantification (L.O.Q.) of plant-derived nanovesicles (PDNVs)**. (A) Correlation between antioxidant activity measured by ORAC and protein content in lemon nanovesicles for laboratory 1, (B) Correlation between antioxidant activity measured by ORAC and protein content in lemon nanovesicles for laboratory 2. (C) L.O.D and L.O.Q. of other PDNVs for laboratory 1 and 2. TEAC, Trolox equivalent antioxidant capacity.

Sample	Laboratory 1			Laboratory 2		
	Observed TEAC	Expected TEAC	Deviation (%)	Observed TEAC	Expected TEAC	Deviation (%)
Hydroxytyrosol (HT)	106.6112			102.2124		
Lemon	16.8388			13.6869		
Melon	3.8200			5.0676		
Blueberry	2.7644			4.4836		
HT+Lemon	113.30	123.4501	8.9603	112.2907	115.8994	3.2136
HT+Melon	107.26	110.4313	2.9568	107.2599	107.2800	0.0187
HT+Blueberry	107.29	109.3757	1.9482	102.1102	106.6960	4.4910

Figure S6. **Spike-in recovery analysis of plant-derived nanovesicles (PDNVs) in the ORAC assay performed in two independent laboratories**. Antioxidant activity was measured for hydroxytyrosol (HT), individual PDNV preparations (lemon, melon, and blueberry), and mixtures containing a fixed amount of HT and each PDNV sample. Antioxidant activity is expressed as Trolox equivalent antioxidant capacity (TEAC; µmol Trolox equivalents/L). Expected values were calculated as the theoretical sum of the antioxidant activities measured for HT and the corresponding PDNV preparation individually, whereas observed values represent the experimentally determined antioxidant activity of the combined samples. Percentage deviation was calculated as the difference between observed and expected values relative to the expected value to evaluate potential PDNV-driven matrix effects. The low percentage deviations (<10%) observed for all PDNV preparations in both laboratories indicate minimal matrix interference in the ORAC assay.

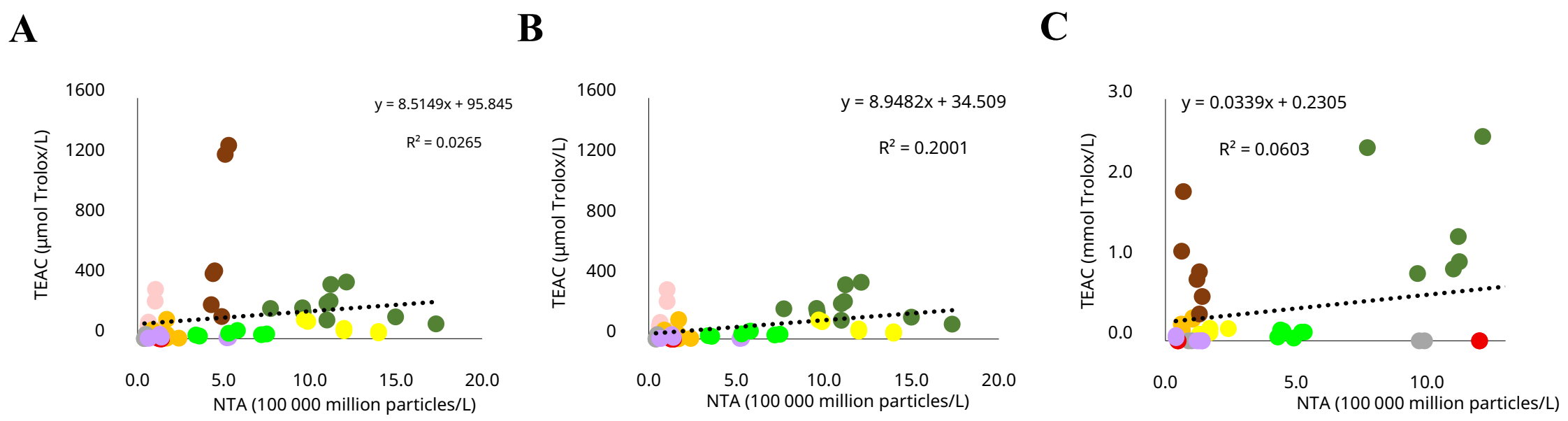

Figure S7. Correlation between EV antioxidant activity and EVs particle number (NTA). (A) Correlation between antioxidant activity measured by ORAC and EV particle number, (B) correlation excluding ginger-derived EVs. (C) Correlation between antioxidant activity measured by ABTS assay and EV particle number. TEAC, Trolox equivalent antioxidant capacity; NTA, nanoparticle tracking analysis.

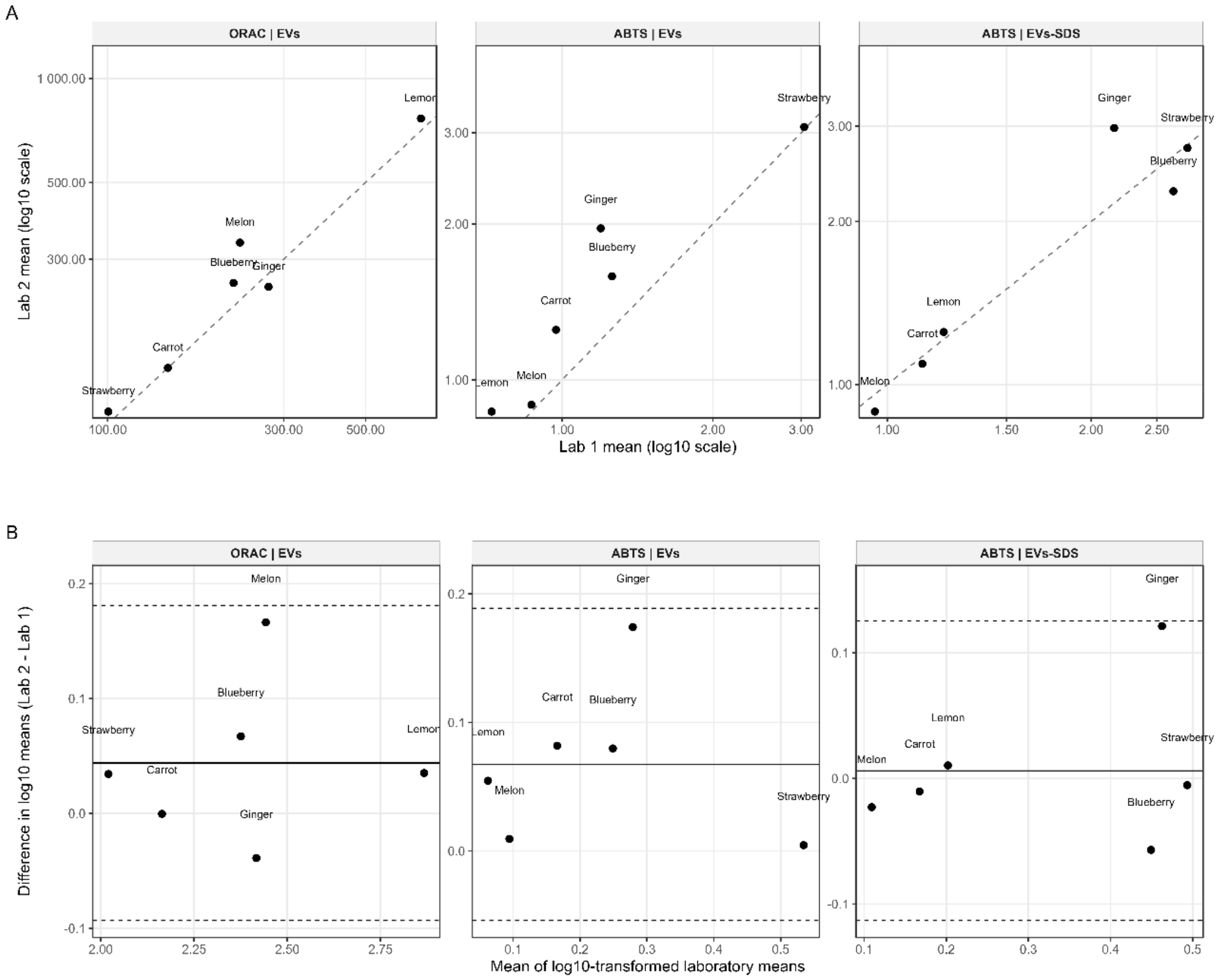

Figure S8. Interlaboratory agreement analysis for ORAC-EVs, ABTS-EVs, and ABTS-EVs-SDS. Panel A shows scatterplots of Lab 1 versus Lab 2 fruit-level means on the log10 scale, with the dashed diagonal indicating the line of identity. Panel B shows Bland–Altman plots based on log10-transformed laboratory means, where the solid horizontal line indicates the mean difference and the dashed lines indicate the limits of agreement. Each point represents one fruit-derived preparation.

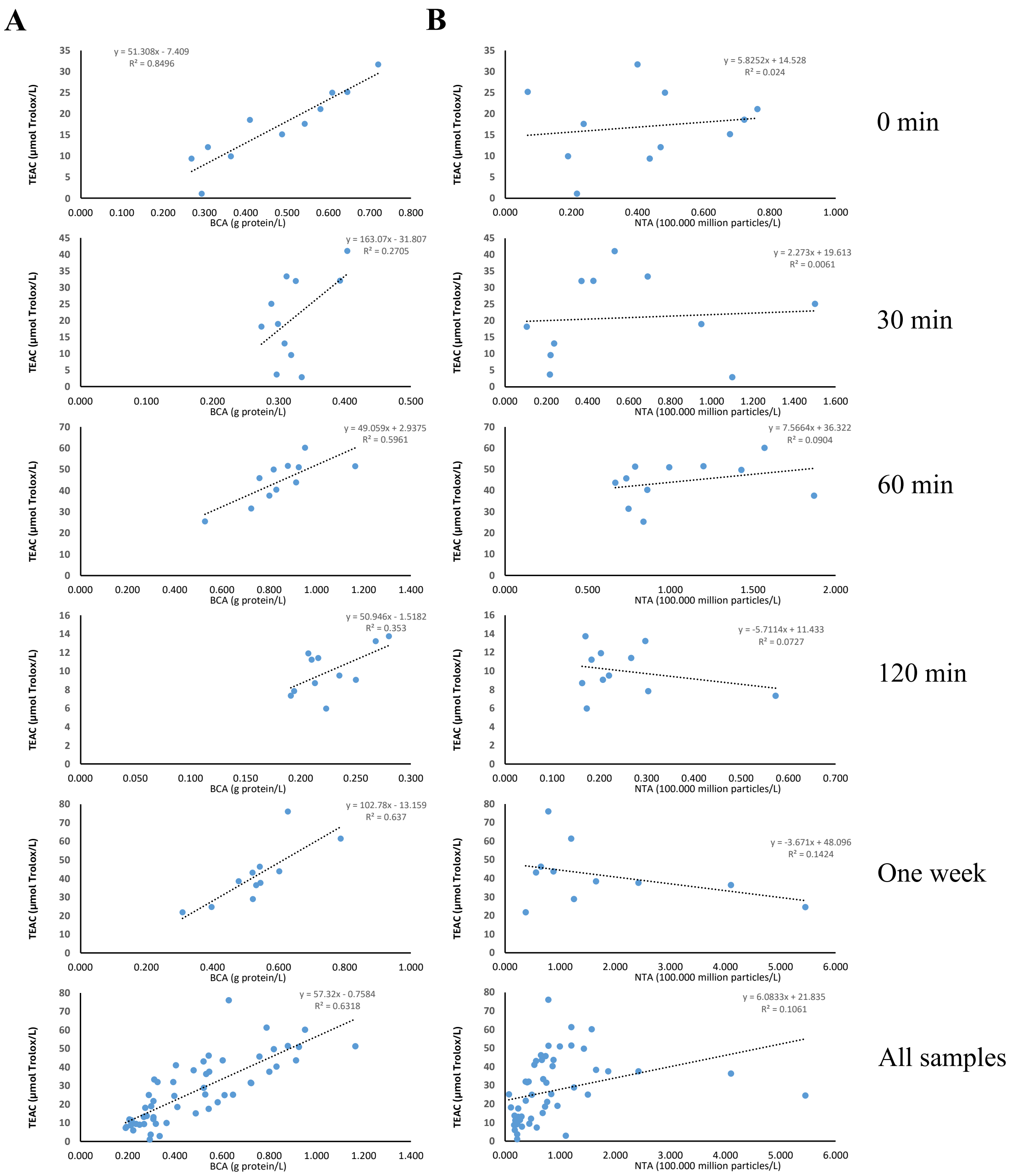

Figure S9. **Correlation between EV antioxidant activity and protein content in mammalian exosomes**. (A) Correlation between antioxidant activity measured by ORAC and EV protein content, (B) Correlation between antioxidant activity measured by ORAC and EV particle number. TEAC, Trolox equivalent antioxidant capacity; NTA, nanoparticle tracking analysis.

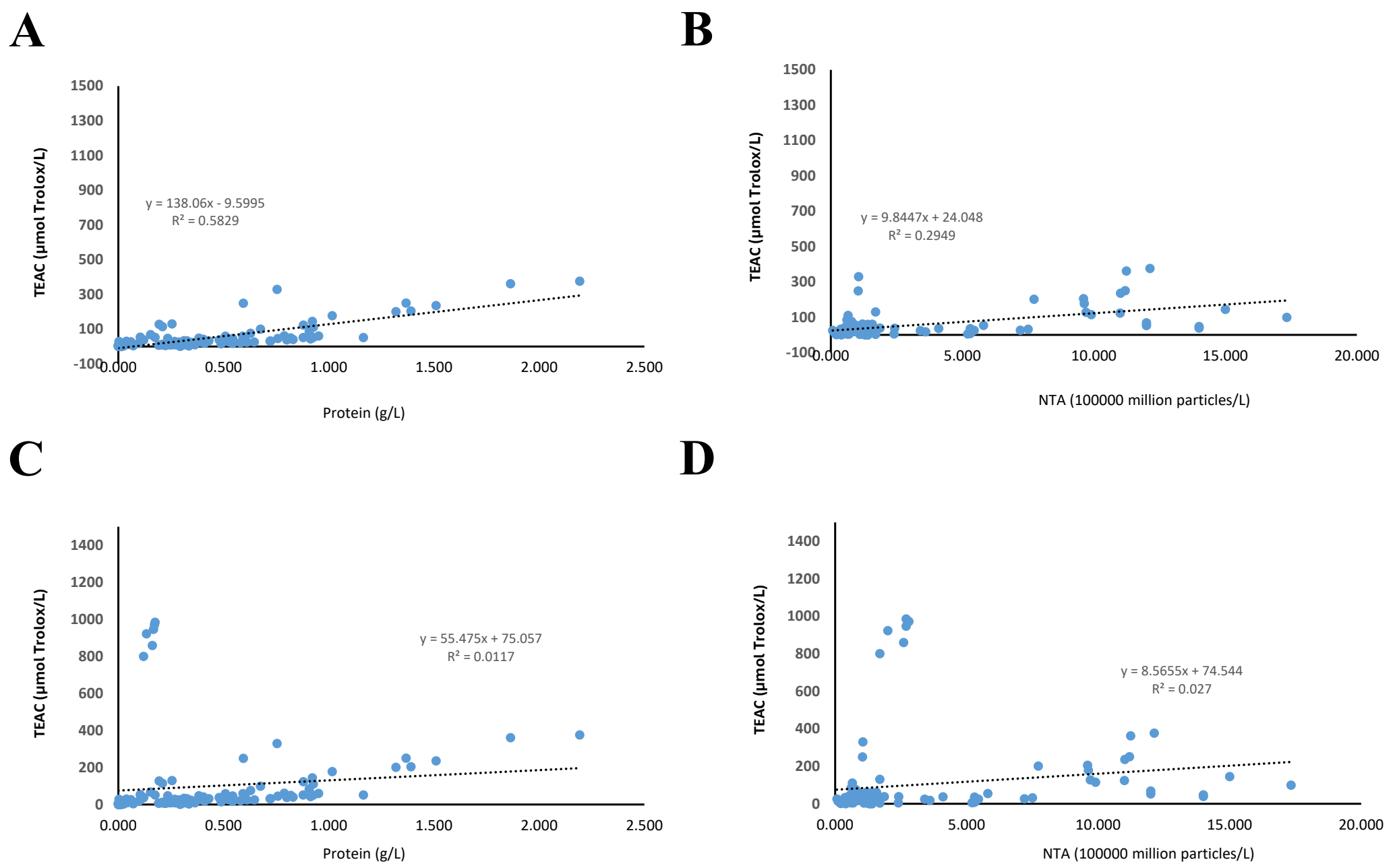

Figure S10. Correlation between antioxidant activity and EV normalization parameters across different biological kingdoms. (A) Correlation between antioxidant activity measured by ORAC (expressed as TEAC) and EV protein concentration for mammalian and plants EVs preparations. (B) Correlation between antioxidant activity measured by ORAC and EV particle concentration determined by nanoparticle tracking analysis (NTA). (C) Correlation between antioxidant activity measured by ORAC and EV protein concentration for all EV preparations (including bacteria). (D) Correlation between antioxidant activity measured by ORAC and EV particle concentration determined by NTA for all EV preparations (including bacteria). Overall, protein content showed a stronger association with antioxidant activity than particle concentration, supporting its use as the preferred normalization parameter for comparisons among evolutionarily related EV populations. TEAC, Trolox equivalent antioxidant capacity; NTA, nanoparticle tracking analysis.

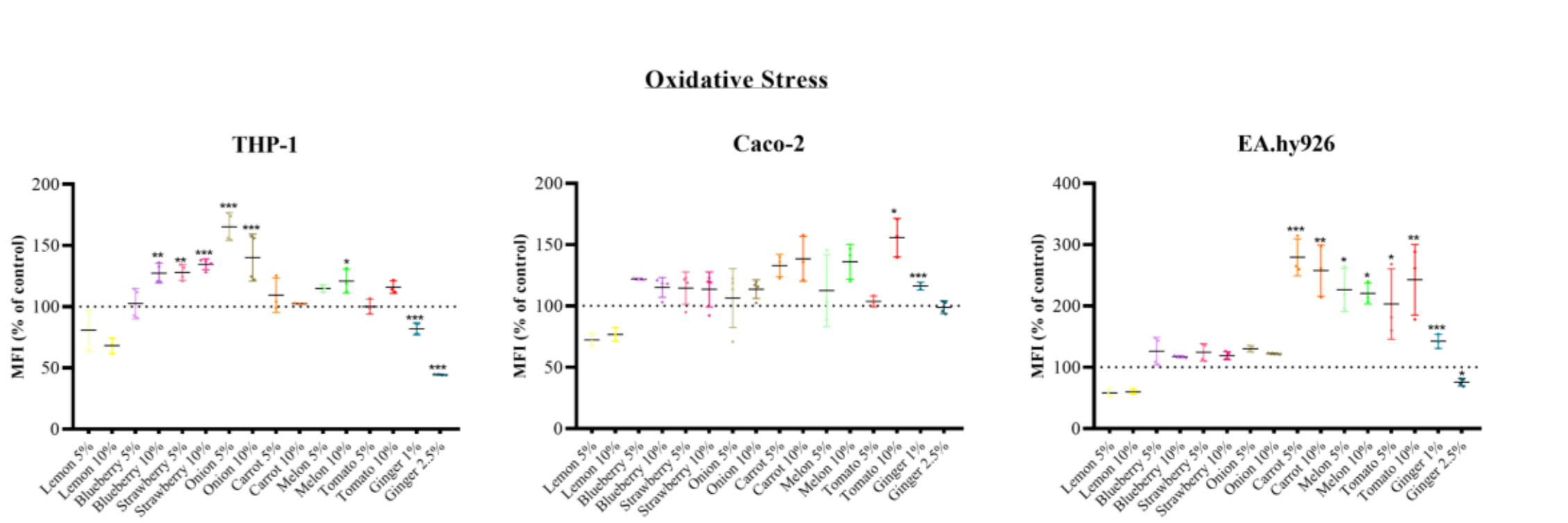

Figure S11. Cell-type-dependent effects of plant-derived nanovesicles on intracellular ROS levels. THP-1 monocytes, EA.hy926 endothelial cells, and Caco-2 intestinal epithelial cells were treated with plant-derived nanovesicles from different botanical sources. Intracellular ROS levels after treatment with PDNVs at the concentrations indicated in panels, expressed as mean fluorescence intensity (MFI) relative to untreated control cells (set to 100%). Data are presented as mean ± SD from at least three independent experiments performed in technical triplicate. Statistical significance was assessed by one-way or two-way ANOVA, as appropriate, followed by Dunnett's post hoc test for multiple comparisons versus control. Significance is indicated by *p < 0.05, **p < 0.01, and ***p < 0.001.

A B

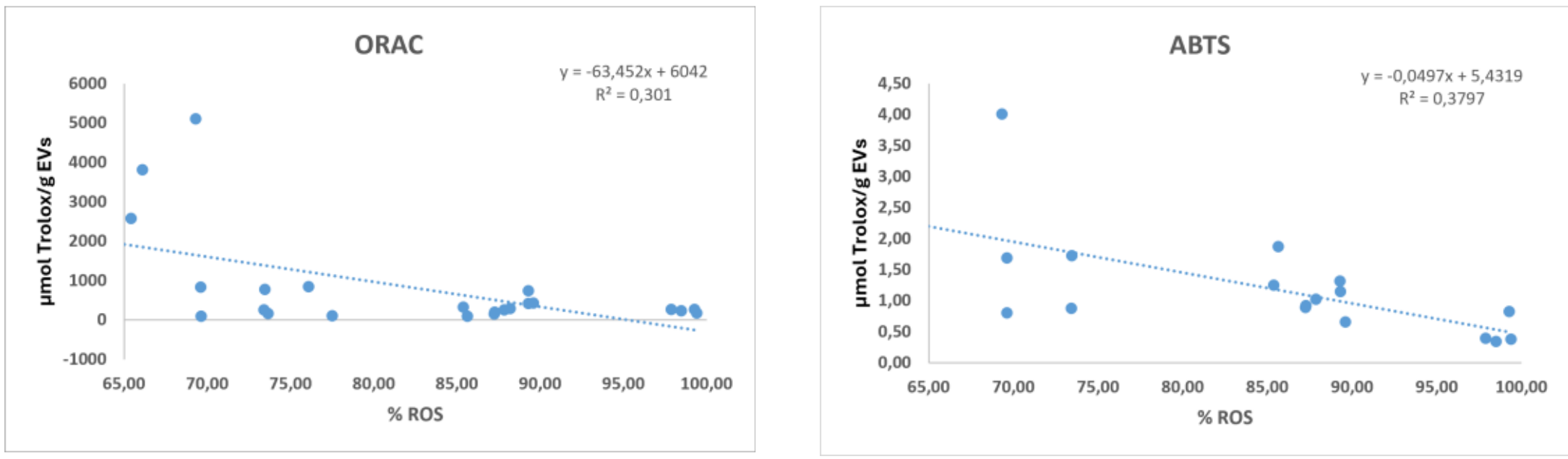

Figure S12. Correlation between chemical antioxidant activity and cellular redox response of purified plant-derived nanovesicles (PDNVs). Correlation analysis between antioxidant activity determined by the ORAC (A) and ABTS (B) assays and intracellular reactive oxygen species (ROS) levels measured in EA.hy926 endothelial cells after 4 h exposure to purified PDNVs derived from different botanical sources. Antioxidant activity is expressed as Trolox equivalent antioxidant capacity (TEAC) normalized to EV protein content (µmol Trolox/g EV protein for ORAC and mmol Trolox/g EV protein for ABTS). Cellular ROS levels are expressed as percentage of the untreated control. Each point represents an individual PDNV preparation. Linear regression analysis and the corresponding coefficient of determination (R^2) are shown. The negative relationship indicates that EV preparations with higher antioxidant capacity measured by the chemical assays generally produced lower intracellular ROS levels, supporting the use of ORAC and ABTS as predictors of the intrinsic antioxidant potential of purified PDNV preparations.

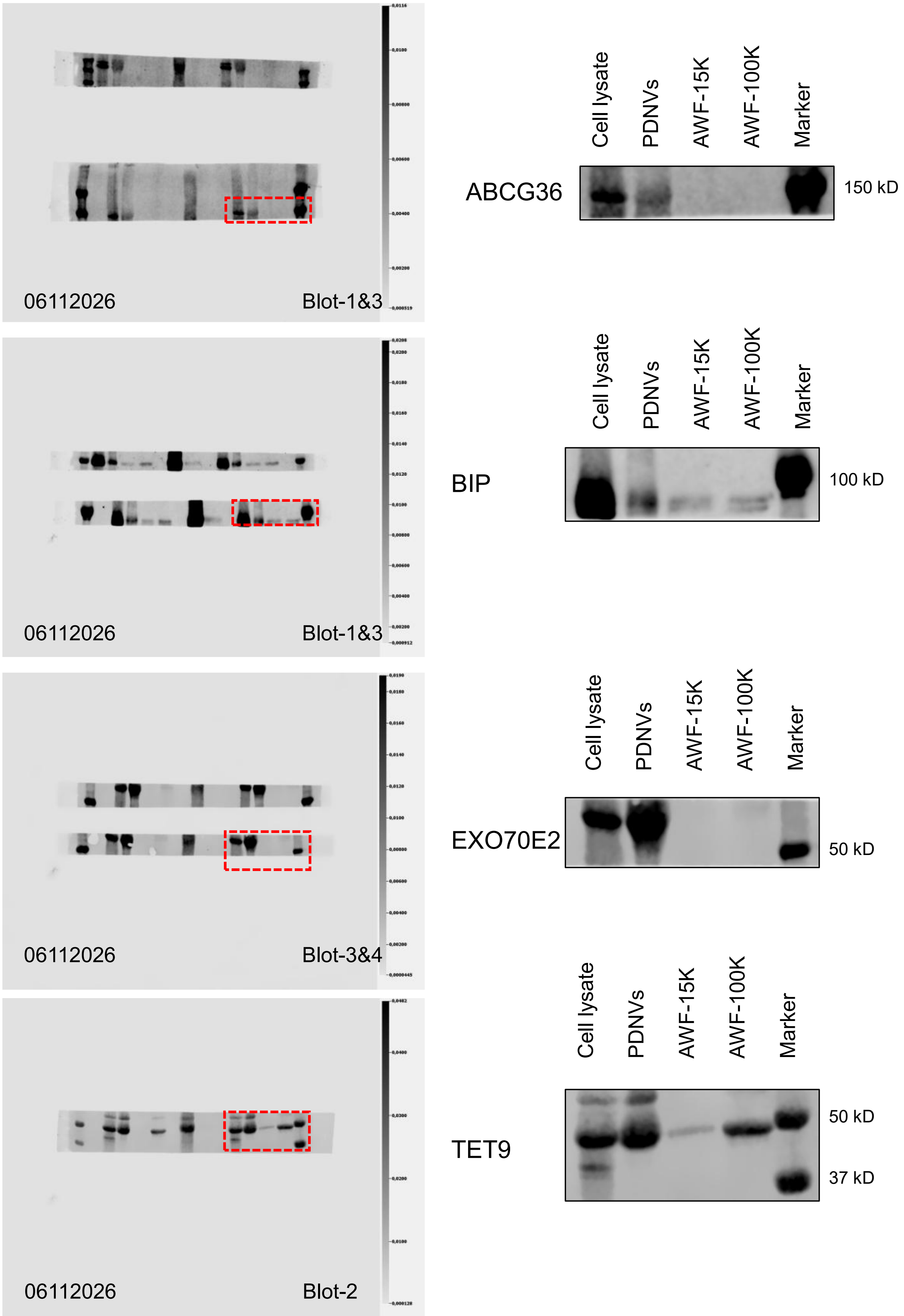
06112026
Blot-1&3
Cell lysate
PDNVs
AWF-15K
AWF-100K
Marker
ABCG36
150 kD
06112026
Blot-1&3
Cell lysate
PDNVs
AWF-15K
AWF-100K
Marker
BIP
100 kD
06112026
Blot-3&4
Cell lysate
PDNVs
AWF-15K
AWF-100K
Marker
EXO70E2
50 kD
06112026
Blot-2
Cell lysate
PDNVs
AWF-15K
AWF-100K
Marker
TET9
50 kD
37 kD

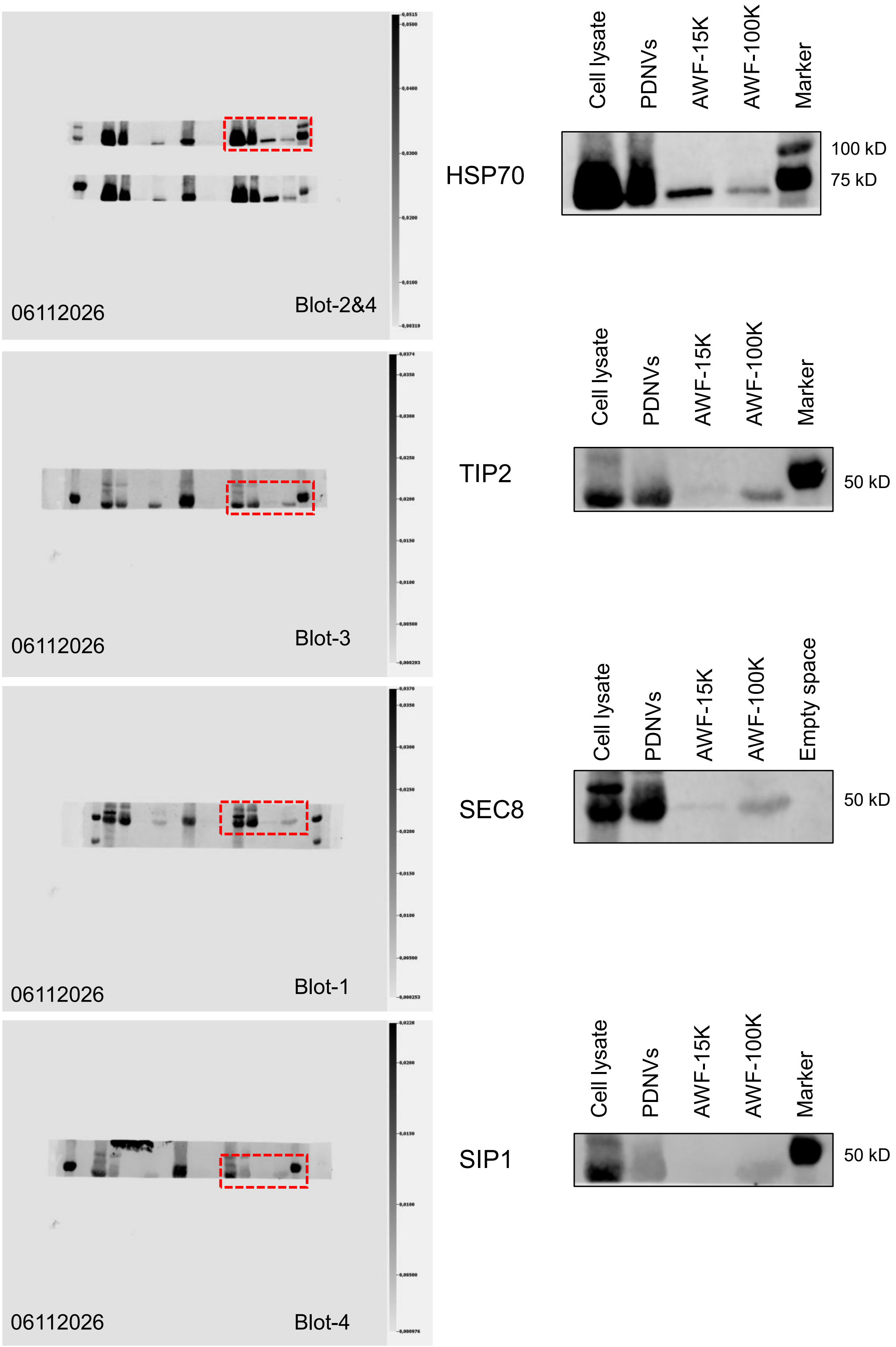
06112026
Blot-2&4
HSP70
Cell lysate
PDNVs
AWF-15K
AWF-100K
Marker
100 kD
75 kD
06112026
Blot-3
TIP2
Cell lysate
PDNVs
AWF-15K
AWF-100K
Marker
50 kD
06112026
Blot-1
SEC8
Cell lysate
PDNVs
AWF-15K
AWF-100K
Empty space
50 kD
06112026
Blot-4
SIP1
Cell lysate
PDNVs
AWF-15K
AWF-100K
Marker
50 kD

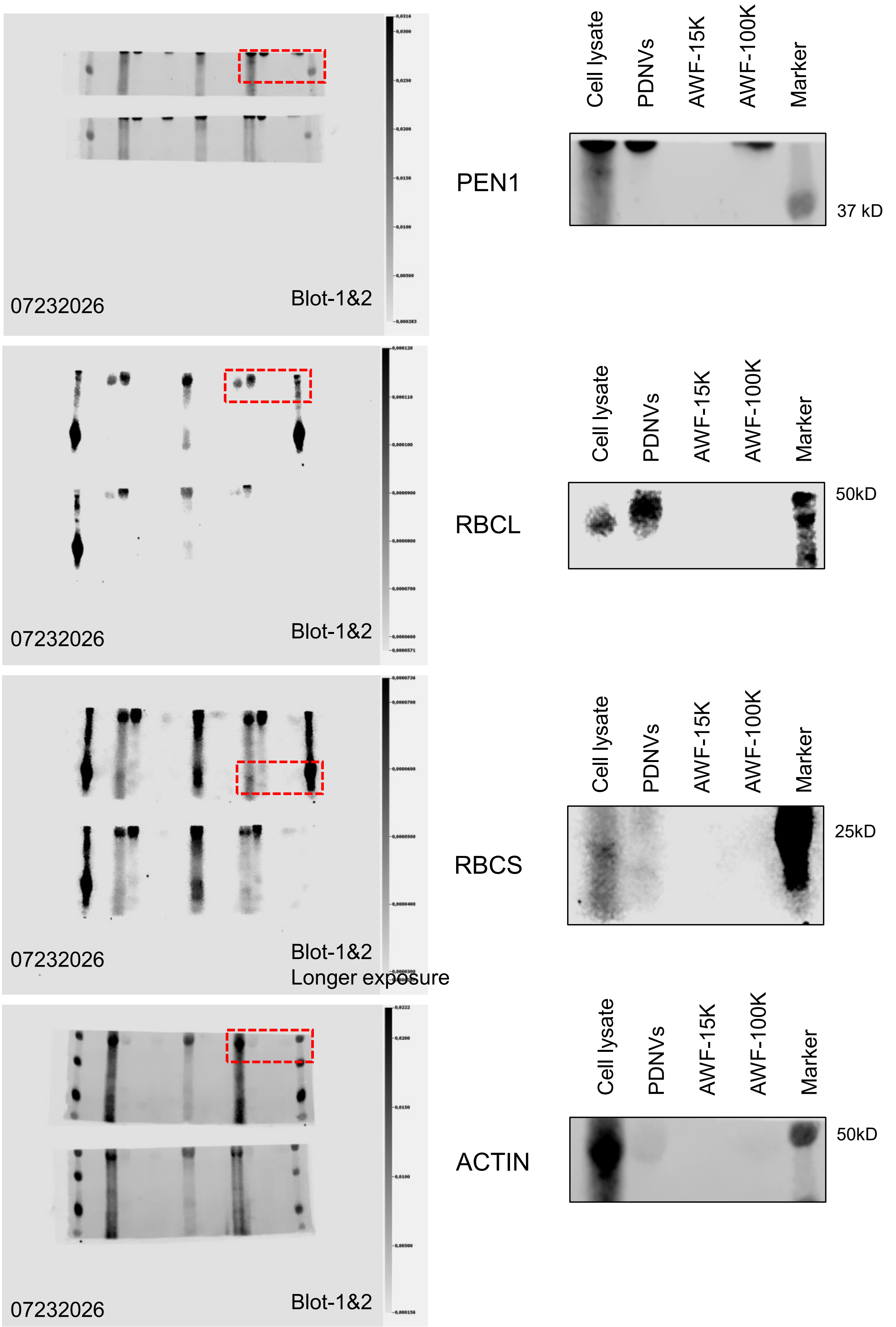

Figure S13. **Full Western blot membrane images corresponding to Figure 4F**. Raw scanned Western blot membrane images corresponding to the cropped Western blot

images presented in Figure 4F of the main manuscript. Crop boundaries are highlighted to indicate the regions used for the corresponding panels in Figure 4F.

Table S1. Complete fruit-level values for n, mean, SD, within-laboratory CV, inter-laboratory CV, laboratory ratios, and absolute/percentage differences.

Assay	Fraction	Fruit	n_Lab1	n_Lab2	mean_Lab1	sd_Lab1	n_lab_cv_pct	in_lab_flag_L	mean_Lab2	sd_Lab2	n_lab_cv_pct	in_lab_flag_L	nterlab_cv_pc	interlab_flag	_over_lab1_r	ff_lab2_minu	ct_diff_vs_lab	ean_log10_di
ORAC	EVs	Strawberry	4	4	100.460302	45.8258761	45.6159049	Fail (>=15%)	108.755339	57.0572092	52.4638235	Fail (>=15%)	5.60711112	Pass (<20%)	1.08257029	8.29503678	8.25702947	0.03433732
ORAC	EVs	Carrot	5	4	145.621672	68.1780415	46.8186092	Fail (>=15%)	145.500687	18.0033924	12.3734072	Pass (<15%)	0.05877211	Pass (<20%)	0.99916918	-0.1209851	-0.0830818	-0.0003601
ORAC	EVs	Lemon	4	3	706.291681	203.969019	28.8788647	Fail (>=15%)	765.961184	243.904489	31.8429307	Fail (>=15%)	5.73172054	Pass (<20%)	1.08448281	59.6695026	8.44828053	0.03520536
ORAC	EVs	Blueberry	3	4	219.400869	157.728904	71.8907382	Fail (>=15%)	256.162763	85.2613067	33.2840361	Fail (>=15%)	10.9321163	Pass (<20%)	1.16755583	36.7618941	16.7555827	0.06717512
ORAC	EVs	Ginger	4	3	272.923791	89.4631055	32.7795188	Fail (>=15%)	249.546384	56.9016889	22.8020491	Fail (>=15%)	6.3277577	Pass (<20%)	0.91434456	-23.377408	-8.565544	-0.0388363
ORAC	EVs	Melon	4	4	228.368133	16.9322169	7.41443946	Pass (<15%)	335.05312	101.752072	30.3689374	Fail (>=15%)	26.7784282	Fail (>=20%)	1.46716232	106.684987	46.7162322	0.16625955
ABTS	EVs	Strawberry	3	3	3.04208469	0.05498116	1.80735126	Pass (<15%)	3.07765691	1.97812867	64.2738528	Fail (>=15%)	0.82203981	Pass (<20%)	1.01169337	0.03557222	1.16933686	0.00451542
ABTS	EVs	Carrot	3	3	0.97220181	0.16635777	17.1114442	Fail (>=15%)	1.24880566	0.24254696	19.4223147	Fail (>=15%)	17.6125892	Pass (<20%)	1.28451279	0.27660385	28.4512795	0.08184937
ABTS	EVs	Lemon	3	3	0.72300765	0.04593017	6.35265312	Pass (<15%)	0.86837285	0.05169611	5.95321597	Pass (<15%)	12.9181826	Pass (<20%)	1.20105624	0.1453652	20.1056242	0.05462741
ABTS	EVs	Blueberry	3	3	1.25755369	0.01643933	1.30724704	Pass (<15%)	1.58399911	0.65223453	41.1764453	Fail (>=15%)	16.2468749	Pass (<20%)	1.25958765	0.32644541	25.9587652	0.07976961
ABTS	EVs	Ginger	3	3	1.19393004	0.38354642	32.124698	Fail (>=15%)	1.96107337	0.38356059	19.558707	Fail (>=15%)	34.3867934	Fail (>=20%)	1.64253625	0.76714333	64.2536252	0.17411861
ABTS	EVs	Melon	3	3	0.86841765	0.00563812	0.64924023	Pass (<15%)	0.89532991	0.64226995	71.7355632	Fail (>=15%)	2.15788763	Pass (<20%)	1.03099	0.02691227	3.09900026	0.00940045
ABTS	EVs-SDS	Strawberry	3	3	2.77284874	0.28956615	10.442912	Pass (<15%)	2.73439194	1.01975923	37.293821	Fail (>=15%)	0.98753878	Pass (<20%)	0.98613094	-0.0384568	-1.386906	-0.0053615
ABTS	EVs-SDS	Carrot	3	3	1.12694447	0.20611596	18.2898063	Fail (>=15%)	1.09182889	0.1482347	13.5767332	Pass (<15%)	2.23821513	Pass (<20%)	0.96884001	-0.0351156	-3.1159986	-0.0103687
ABTS	EVs-SDS	Lemon	3	3	1.21168926	0.09067236	7.4831367	Pass (<15%)	1.24982059	0.28258899	22.6103642	Fail (>=15%)	2.19076278	Pass (<20%)	1.03146956	0.03813133	3.14695604	0.01040096
ABTS	EVs-SDS	Blueberry	3	3	2.64356409	1.06705816	40.3643766	Fail (>=15%)	2.27446883	0.04531251	1.99222405	Pass (<15%)	10.6135834	Pass (<20%)	0.86037968	-0.3690953	-13.962032	-0.0569134
ABTS	EVs-SDS	Ginger	3	3	2.16210514	0.59217306	27.3887265	Fail (>=15%)	2.97538496	0.65794877	22.1130636	Fail (>=15%)	22.3874175	Fail (>=20%)	1.37615184	0.81327982	37.6151839	0.12131967
ABTS	EVs-SDS	Melon	3	3	0.95869122	0.24149998	25.1905909	Fail (>=15%)	0.89100425	0.0122348	1.37314765	Pass (<15%)	5.17511259	Pass (<20%)	0.92939649	-0.067687	-7.0603509	-0.0228576

*See Legend (below) for column definitions and calculation notes.

**Column name**	**Meaning**	**How calculated / interpretation**
n_Lab1	Replicate count in Lab 1	Number of replicate measurements used in Lab 1
n_Lab2	Replicate count in Lab 2	Number of replicate measurements used in Lab 2
mean_Lab1	Mean value in Lab 1	Average of replicate values from Lab 1
sd_Lab1	Standard deviation in Lab 1	SD of replicate values from Lab 1
within_lab_cv_pct_Lab1	Within-laboratory CV (%) in Lab 1	100 × SD / mean using replicate values from Lab 1 only
within_lab_flag_Lab1	Within-laboratory repeatability flag for Lab 1	Pass if CV < 15%; Fail if CV ≥ 15%
mean_Lab2	Mean value in Lab 2	Average of replicate values from Lab 2
sd_Lab2	Standard deviation in Lab 2	SD of replicate values from Lab 2
within_lab_cv_pct_Lab2	Within-laboratory CV (%) in Lab 2	100 × SD / mean using replicate values from Lab 2 only
within_lab_flag_Lab2	Within-laboratory repeatability flag for Lab 2	Pass if CV < 15%; Fail if CV ≥ 15%
interlab_cv_pct	Inter-laboratory CV (%)	100 × SD / mean using the Lab 1 mean and Lab 2 mean
interlab_flag	Inter-laboratory agreement flag	Pass if inter-laboratory CV < 20%; Fail if CV ≥ 20%
lab2_over_lab1_ratio	Lab 2 / Lab 1 ratio	Mean_Lab2 divided by mean_Lab1
abs_diff_lab2_minus_lab1	Absolute difference between laboratories	Mean_Lab2 − mean_Lab1
pct_diff_vs_lab1	Percentage difference relative to Lab 1	100 × (Mean_Lab2 − Mean_Lab1) / Mean_Lab1
Abbreviation	Definition	
W-L CV	Within-laboratory coefficient of variation	
I-L CV	Inter-laboratory coefficient of variation	
